# Immune Checkpoints in B Cells: Unlocking New Potentials in Cancer Treatment

**DOI:** 10.1002/advs.202403423

**Published:** 2024-11-07

**Authors:** Xiaoye Shi, Xiangshu Cheng, Aimin Jiang, Wenjie Shi, Lingxuan Zhu, Weiming Mou, Antonino Glaviano, Zaoqu Liu, Quan Cheng, Anqi Lin, Linhui Wang, Peng Luo

**Affiliations:** ^1^ Department of Oncology Zhujiang Hospital Southern Medical University Guangzhou Guangdong 510282 China; ^2^ The Second School of Clinical Medicine Southern Medical University Guangzhou Guangdong 510515 China; ^3^ College of Bioinformatics Science and Technology Harbin Medical University 157 Baojian Road. Nangang District, Harbin Heilongiiang 150076 China; ^4^ Department of Urology Changhai Hospital Naval Medical University (Second Military Medical University) Shanghai 200433 China; ^5^ Molecular and Experimental Surgery University Clinic for General‐ Visceral‐ Vascular‐ and Trans‐Plantation Surgery Medical Faculty University Hospital Magdeburg Otto‐von Guericke University 39120 Magdeburg Germany; ^6^ Department of Urology Shanghai General Hospital Shanghai Jiao Tong University School of Medicine Shanghai 200080 China; ^7^ Department of Biological Chemical and Pharmaceutical Sciences and Technologies University of Palermo Palermo 90123 Italy; ^8^ Institute of Basic Medical Sciences Chinese Academy of Medical Sciences and Peking Union Medical College Beijing 100730 China; ^9^ Department of Neurosurgery Xiangya Hospital Central South University Changsha 410008 China; ^10^ National Clinical Research Center for Geriatric Disorders Xiangya Hospital Central South University Changsha 410008 China; ^11^ Cancer Centre and Institute of Translational Medicine Faculty of Health Sciences University of Macau Macau SAR 999078 China

**Keywords:** antitumor immunity, B cell, immune checkpoint (IC), immune checkpoint inhibitor (ICI), tumor immune microenvironment (TME)

## Abstract

B cells are crucial component of humoral immunity, and their role in the tumor immune microenvironment (TME) has garnered significant attention in recent years. These cells hold great potential and application prospects in the field of tumor immunotherapy. Research has demonstrated that the TME can remodel various B cell functions, including proliferation, differentiation, antigen presentation, and antibody production, thereby invalidating the anti‐tumor effects of B cells. Concurrently, numerous immune checkpoints (ICs) on the surface of B cells are upregulated. Aberrant B‐cell IC signals not only impair the function of B cells themselves, but also modulate the tumor‐killing effects of other immune cells, ultimately fostering an immunosuppressive TME and facilitating tumor immune escape. Blocking ICs on B cells is beneficial for reversing the immunosuppressive TME and restoring anti‐tumor immune responses. In this paper, the intricate connection between B‐cell ICs and the TME is delved into, emphasizing the critical role of targeting B‐cell ICs in anti‐tumor immunity, which may provide valuable insights for the future development of tumor immunotherapy based on B cells.

## Graphical abstract

Recent studies have identified B‐cell tumor‐specific immune checkpoints (ICs). This review elucidates the dysfunction of B cells in tumor microenvironment, summarizes the discovery and mechanism of action of B cell ICs in tumors, and discusses urgent issues and future perspectives.

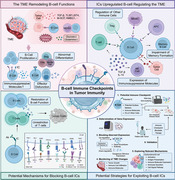

## 1 Introduction

B cells are a major component of humoral immunity in the body, and their role in tumor immunity has been continuously and intensively studied.^[^ ^1^ ^]^ Some studies have shown that regulatory B cells (Bregs) in the tumor microenvironment (TME) tend to secrete inflammatory cytokines such as Interleukin (IL) ‐10 and transforming growth factor‐β (TGF‐β) to promote tumor progression.^[^ ^2^, ^3^, ^4^ ^]^ In recent years, the anti‐tumor effect of B cells has also been gradually discovered.^[^ ^5^ ^]^ B cells can produce specific antibodies that recognize and bind tumor neoantigens generated by mutations or abnormal post‐transcriptional modifications.^[^ ^6^, ^7^ ^]^ Moreover, activated B cells maintain or enhance T‐cell anti‐tumor immune responses through antigen presentation and co‐stimulation.^[^ ^8^, ^9^, ^10^ ^]^ In some tumor microenvironments, B cells construct local antitumor immune responses in the form of the tertiary lymphoid structure(TLS).^[^ ^11^, ^12^ ^]^ A spatial transcriptomic analysis demonstrated the presence of a B‐cell response, including B‐cell maturation and antibody production in the intratumoral TLS in renal cell carcinoma (RCC).^[^ ^13^ ^]^ Similar results were found in pancreatic ductal adenocarcinoma, melanoma, and other cancers.^[^ ^14^, ^15^ ^]^ Thus, B cells are involved in complex immune responses in the TME and have become one of the new targets for tumor therapy.

An immune checkpoint (IC) is a braking molecule designed to prevent over‐activation of the immune system, and the discovery and application of ICs in tumors has opened a new chapter in tumor therapy.^[^ ^16^, ^17^, ^18^ ^]^ Cytotoxic T lymphocyte associate protein‐4(CTLA‐4) was the earliest IC to be identified.^[^ ^19^ ^]^ Utilizing a CTLA‐4 knockout mouse model, researchers have demonstrated that CTLA‐4 on the surface of T‐cells inhibits T‐cell activation and exerts negative regulatory effects.^[^ ^19^ ^]^ In addition to T cells, there has been much interest in tumor ICs of natural killer (NK) cells and macrophages.^[^ ^20^, ^21^ ^]^ For example, tumor‐infiltrating T cell immunoreceptor with immunoglobulin and ITIM domain(TIGIT), which is highly expressed on NK cells, has been associated with NK cell exhaustion.^[^ ^22^ ^]^ Blocking TIGIT reverses NK cell exhaustion and enhances adaptive antitumor immunity.^[^ ^22^ ^]^ Moreover, recombinant sialic acid binding Ig like lectin 9 (Siglec‐9) has been shown to be an immune checkpoint molecule expressed on macrophages with a role in restricting the antitumor response of T cells.^[^ ^23^ ^]^ Blocking aberrant expression or function of ICs facilitates the restoration of immune cells' tumor‐killing function and reversing the immunosuppressive microenvironment.^[^ ^18^ ^]^ The immune checkpoint inhibitors (ICIs) have been widely used in the clinic, showing good therapeutic efficacy and improving the survival of tumor patients.^[^ ^24^, ^25^ ^]^

In recent years, tumor ICs of B cells have also been gradually identified. A recent study identified an upregulation of T cell immunoglobulin and mucin domain‐containing protein(TIM‐1) expression in B cells and limited the proliferative and tumor‐killing effects of T cells.^[^ ^26^ ^]^ Blockade of TIM‐1 expression lowered the B‐cell receptor (BCR) activation threshold while enhancing the antigen presentation and co‐stimulatory functions of B cells.^[^ ^26^ ^]^ A number of previous studies have also characterized the expression of tumor‐associated ICs on B cells, including CTLA‐4,^[^ ^27^ ^]^ TIGIT,^[^ ^28^ ^]^ PD‐L1,^[^ ^29^, ^30^ ^]^ and others. These aberrantly upregulated IC signals contribute significantly for B cells to undergo dysfunction and facilitate tumor immune escape. Thus, blocking the aberrant expression of IC in B cells is crucial important for suppressing tumor progression.

However, the potential of B‐cell ICs in tumor immunotherapy has not been fully explored. Compared with T cells, the study of B‐cell ICs in tumors is still in its infancy, with numerous unresolved issues that require further investigation, such as an in‐depth exploration of B‐cell ICs, the relationship between B‐cell ICs, B cells, and TME, the development and clinical validation of B‐cell ICIs. Considering the intricate and multifaceted roles of B cells and their IC expression within the TME, a comprehensive investigation of B cell dysfunction and aberrant IC expression in the TME could offer theoretical foundations for identifying additional B cell ICs and developing novel tumor immunotherapeutic strategies targeting B cell ICs, ultimately leading to improved therapeutic efficacy and clinical outcomes for tumor patients.

ICs expressed on B cells have gradually shown promise as potential tumor immunotherapeutic targets.^[^ ^31^ ^]^ However, there is no review systematically summarizing the research progress of B cell ICs in tumors. In this paper, we elucidated B cell dysfunction within the TME, summarized the discovery and mechanism of action of B cell IC in tumors, and elaborated on the possible mechanisms of targeting B cell IC to restore anti‐tumor immune effects. In addition, we discussed the urgent issues and future research directions for tumor‐associated B‐cell ICs and proposed potential strategies for B‐cell IC development. We hoped that this paper will lay a theoretical foundation and provide valuable insights for the further development of tumor ICs of B cells and their application in tumor immunotherapy.

## 2 B Cells' Dysfunction in TME

**Figure 1:**
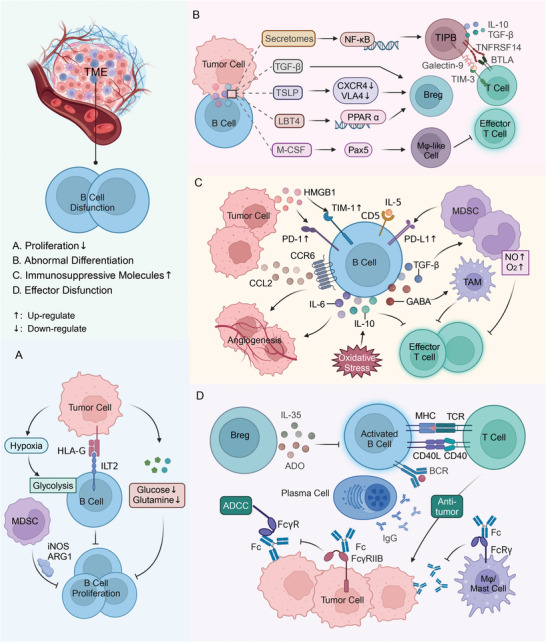
Dysfunction of B cells in the TME. A) Tumor cells block B cell proliferation by high expression of HLA‐G and predation of oxygen and glucose in the TME, and MSDCs can also inhibit B cell proliferation through specific molecules. B) Tumor cells can modulate specific immune pathways, causing B cells to differentiate into TIPBs, Bregs, and macrophage‐like cells, and these types of B cells are detrimental to anti‐tumor immune responses. C) Tumor cells and MDSCs upregulate ICs on the surface of B cells, while IL‐5 and oxidative stress in the TME promote the expression of inflammatory factors, such as IL‐6, IL‐10, and TGF‐β, in B cells, and these immunosuppressive molecules negatively regulate effector T cells and promote tumor progression. D) ICs upregulated in B cells can limit the activation, antigen presentation, and co‐stimulatory functions of B cells and affect the anti‐tumor effect of T cells. Moreover, antibodies in the TME can bind to tumor cells and macrophages, mast cells, etc. without being able to participate in the tumor killing effect. Abbreviations: TME, tumor immune microenvironment; IL, Interleukin; MDSC, myeloid‐derived suppressor cell; HLA, human histocompatibility leukocyte antigen; ILT2, immunoglobulin‐like transcript 2; iNOS, inducible nitric oxide synthase; ARG1, arginase 1; TIPB, tumor‐induced plasmablast‐like‐enriched B cell; IL, Interleukin; TGF‐β, transforming growth factor‐β; PPARα, peroxisome proliferator‐activated receptor α; Breg, regulatory B cell; TSLP, thymic stromal lymphopoietin; LTB4, leukotriene B4; TSLP, thymic stromal lymphopoietin; Mφ, Macrophage; TAM, tumor‐associated macrophage; BCR, B‐cell receptor; TAB, tumor‐associated B cell; IFN, interferon; ADO, adenosine; SHP‐2, src homology 2‐domain‐containing tyrosine phosphatase 2 to phosphotyrosine; ADCC, antibody‐dependent cell‐mediated cytotoxicity. This figure was created based on the tools provided by Biorender.com.

In recent years, studies have shown that a dysregulated TME can affect the normal function of B cells, including B cell proliferation, differentiation, immune molecule expression, and effector functions, ultimately promoting tumor immune escape (**Figure** **1**).^[^ ^32^, ^33^ ^]^

### 2.1 TME Inhibits B Cells’ Proliferation

Several components of the TME, including tumor cells, immune cells, immune molecules, and non‐immune components, can affect B cell proliferation (Figure 1A). Human leukocyte antigen (HLA)‐G, high‐expressing in some tumors, exerts an inhibitory effect on B cells' proliferation when it binds to immunoglobulin‐like transcript 2 (ILT2) on the surface of the B cells.^[^ ^34^, ^35^, ^36^ ^]^ In addition, the enormous oxygen consumption of tumor cells often leads to hypoxia in the TME.^[^ ^37^ ^]^ In hypoxic TME, B‐cell glycolysis is increased, which is detrimental to B‐cell proliferation and promotes B‐cell apoptosis.^[^ ^38^, ^39^ ^]^ Glucose and glutamine, which are an important source of energy for B cells and maintain B‐cell growth, are often depleted in the TME.^[^ ^40^ ^]^ Localized nutrient deficiencies in the TME make B‐cell proliferation inhibited. Myeloid‐derived suppressor cells (MDSCs) are the currently identified immune cells that inhibit B‐cell proliferation in the TME.^[^ ^41^ ^]^ In a mouse model of lung cancer, MDSCs inhibit B‐cell proliferation through inducible nitric oxide synthase (iNOS) and arginase 1 (ARG1).^[^ ^41^ ^]^ Several previous studies have also demonstrated a negative regulatory effect of MDSCs on B‐cell proliferation.^[^ ^41^, ^42^, ^43^ ^]^ As an important immunosuppressive molecule, TGF‐β has also been suggested to potentially impair B‐cell proliferation.^[^ ^44^, ^45^ ^]^

### 2.2 TME Promotes B Cells' Abnormal Differentiation

To evade immune surveillance, tumor cells can induce aberrant differentiation of B cells through their secretomes, interfering with the immune response to the local TME (Figure 1B). For example, melanoma secretomes activate NF‐κB in tumor‐associated B cells (TABs), which in turn induces the differentiation of TABs into tumor‐induced plasmablast‐like‐enriched B cells (TIPBs).^[^ ^32^ ^]^ The inhibitory ligands on the surface of TIPB, Galectin‐9, and TNFRSF14 are upregulated, which bind to TIM‐3 and BTLA on the surface of T cells. TIPBs also secretes the immunosuppressive cytokines IL‐10 and TGF‐β, which results in T cell dysfunction and suppression of anti‐tumor immune responses.^[^ ^32^ ^]^

Immune‐related molecules secreted by tumor cells also contribute to the aberrant differentiation of B cells and promote immune escape. In a mouse model of breast cancer, tumor cells use their production of leukotriene B4 (LTB4) to activate peroxisome proliferator‐activated receptor α (PPARα) on B cells, which results in the induction of differentiation of tumor‐evoked regulatory B cells (tBregs).^[^ ^33^ ^]^ Deletion of the PPARα gene on B cells inhibits the tBregs generation. TGF‐β is also one of the common molecules secreted by tumor cells, which induces immunosuppressive Bregs, leading to accelerated tumor progression.^[^ ^46^ ^]^ Thymic stromal lymphopoietin (TSLP) secreted by tumor cells down‐regulates the expression of CXCR4 and VLA4 in B‐cell precursors, which, in turn, promotes premature migration out of the bone marrow and the proliferation and differentiation of B‐cell precursors into Bregs that have tumor‐promoting metastatic effects.^[^ ^47^ ^]^ In contrast, tumor metastasis is inhibited when B‐cell CXCR4 and VLA4 expression is downregulated. M‐CSF is another substance secreted by tumor cells that inhibits the normal differentiation of B‐cells.^[^ ^48^ ^]^ Tumor‐derived M‐CSF promotes the downregulation of Pax5 expression in CSF1R + immature B‐cells, which leads to macrophage‐like cell differentiation.^[^ ^48^ ^]^ These macrophage‐like cells have a role in inhibiting effector T cell proliferation and promoting tumor infiltration.

Therefore, in the presence of tumor‐derived molecules, B cells differentiate abnormally into TIPBs, macrophage‐like cells, and tBregs, which is detrimental to the antitumor immune response.

### 2.3 TME Promotes B Cells' Expression of Immunosuppressive Molecules

Current evidence suggests that tumor cells and MDSCs in the TME promote the expression of IC molecules on B cells (Figure 1C). For example, hepatocellular carcinoma (HCC)‐derived exosomes highly express HMGB1, and HMGB1 strongly induces B cells to express TIM‐1 through the TLR2/4‐MAPK pathway.^[^ ^49^ ^]^ These TIM‐1+ B cells correlate with poor clinical prognosis in HCC. Hepatocellular carcinoma cell‐derived soluble factors upregulate TLR4‐mediated B‐cell lymphoma 6 (BCL6), and BCL6 has an important role in promoting PD‐1 expression in B cells.^[^ ^50^ ^]^ Besides, researchers have found that MDSC‐activated B cells in a breast cancer animal model highly express PD‐L1, and this class of PD‐1‐PD‐L1+CD19+ B cells was defined as a novel class of regulatory B‐cells.^[^ ^51^ ^]^ The number of these Bregs was also positively correlated with poor prognosis of breast cancer.^[^ ^52^ ^]^ The mechanism by which MDSCs regulates B‐cells may be the activation of the PI3K/AKT/NF‐κB pathway.^[^ ^52^ ^]^ Blockade of PD‐1/PD‐L1 or PI3K/AKT signaling reversed PD‐1‐PD‐L1+CD19+ Bregs immunosuppression as well as tumor growth. The role of upregulated expression of B‐cell ICs in tumor immunity will be discussed in detail below.

In the TME, B cells also express or secrete other immunomodulatory molecules of non‐IC nature. IL‐5 in TME binds to CD5 on the surface of B cells and activates STAT3 signaling through gp130 and JAK2, while positively feedback promoting CD5 expression.^[^ ^53^ ^]^ CD5+ B cells are associated with poor tumor progression.^[^ ^53^ ^]^ Tumor‐associated oxidative stress activates ten‐eleven translocation‐2 (TET2) to promote B cells to secrete IL‐10.^[^ ^54^ ^]^ IL‐10‐expressing B cells have been found to inhibit the function of effector T cells and limit antitumor immunity.^[^ ^55^, ^56^ ^]^ Furthermore, in hepatocellular carcinoma tissues, B cells overexpress CCR6 and interact with tumor‐cell‐derived CCL20 to enhance angiogenesis and promote tumor progression.^[^ ^57^ ^]^ It has been found that GABA produced by B cells can induce the differentiation of tumor‐associated macrophages with inhibitory CD8+ T‐cell killing effects against tumor immunity.^[^ ^58^ ^]^ In melanoma, IL‐6 derived from circulating B cells not only directly inhibits apoptosis and promotes tumor angiogenesis, but also activates the transcription factor c‐Maf in T cells to exhaust CD4 and CD8 T cells.^[^ ^59^ ^]^ In addition, through a partial dependence on TGF‐β R1/2 signaling, tumor‐induced upregulation of TGF‐β expression in B cells activates MDSCs, which indirectly promotes tumor escape and metastasis.^[^ ^60^ ^]^ Moreover, these MDSCs will also potentiate the inhibitory effects on CD4+ T cells and CD8+ T cells by promoting the production of reactive oxygen species and nitric oxide in the TME to further promote tumor progression.

### 2.4 TME Promotes B Cells' Effector Dysfunction

In the TME, B cells exert antitumor immune functions through antigen presentation and antibody production. B cells activated by tumor‐associated antigens present tumor antigens to T cells via major histocompatibility complex (MHC) I or MHC II molecules, thereby facilitating T cell‐mediated tumor cytotoxicity.^[^ ^8^, ^61^, ^62^ ^]^ Moreover, these activated B cells can also proliferate and differentiate into plasma cells, also known as effector B cells, which produce tumor‐specific antibodies.^[^ ^61^, ^62^, ^63^ ^]^ Tumor‐specific antibodies can directly eliminate tumor cells through antibody‐dependent cell‐mediated cytotoxicity (ADCC) or complement‐dependent cytotoxicity (CDC).^[^ ^6^, ^7^ ^]^

However, the TME can negatively regulate antibody production by B cells, potentially impairing their antitumor effects (Figure 1D). In head and neck cancer, adenosine (ADO)‐producing Bregs have been identified.^[^ ^64^ ^]^ These extracellular ADOs bind to their corresponding receptors on effector B cells and inhibit intracellular Bruton's tyrosine kinase (BTK) and Ca^2+^ influx, which leads to aberrant BCR signaling and limiting B cell activation and antibody production.^[^ ^64^ ^]^ In addition, in pancreatic cancer, IL‐35 produced by Bregs upregulates BCL6, a transcriptional regulator in naive B cells, through stimulation of the STAT3‐PAX5 complex. This results in dysregulation of the transcriptional program, inhibiting the differentiation of naive B cells into antitumor plasma cells and ultimately leading to B cell effector dysfunction.^[^ ^65^ ^]^

Existing studies suggest that there may be a link between antibody production by B cells and immune escape. Some scholars suggest that tumor‐specific antibodies bind tumor cells with their Fab portion, while the inhibitory Fcγ receptor FcγRIIB of tumor cells captures the Fc portion of the antibody, leading to the inability of the FcγR of effector cells to recognize the Fc portion of the antibody, ultimately escaping from humoral immunity.^[^ ^66^, ^67^, ^68^ ^]^ Besides, in human papillomavirus (HPV)‐16 squamous cell carcinoma, the IgG antibodies secreted by plasma cells in the form of immune complexes are deposited at the tumor site and bind to the Fc receptor common γ chain (FcRγ) on macrophages and mast cells, thereby blocking the contact of the antibody with the tumor cells and killing tumor cell.^[^ ^69^ ^]^

## 3 Functional Characterization of B Cells with High IC Expression in TME

**Figure 2:**
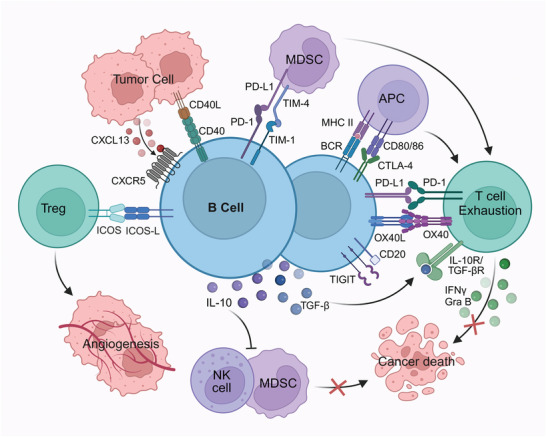
Mechanism of tumor immune response restriction by B cells with high IC expression. IC molecules such as TIM‐1, CTLA‐4, PD‐1, TIGIT, and other IC molecules highly expressed on the surface of B cells inhibit T cell proliferation and expression of IFN‐γ, and promote the exhaustion of T cells; B cells with upregulated expression of IC also regulate the functions of NK cells, MDSC, and Treg, and indirectly inhibit the anti‐tumor response. In addition, B cells with upregulated expression of IC express the immunosuppressive molecules IL‐10 and TGF‐β and act on T cells and other immune cells, which further enhance the inhibition of tumor killing. Abbreviations: MDSC, myeloid‐derived suppressor cell; BCR, B‐cell receptor; APC, antigen‐presenting cell; IL, Interleukin; TGF‐β, transforming growth factor‐β; Gra B, Granzyme B; NK, natural killer cell; IFN, interferon. This figure was created based on the tools provided by Biorender.com.

In the context of tumorigenesis and tumor development, B cells often inhibit anti‐tumor immune responses through IC signaling, thereby promoting tumor progression.^[^ ^26^ ^]^ These IC signals regulate CD4+ T cells, CD8+ T cells, and regulatory T cells (Tregs), promote the release of IL‐10 from B cells and influence the functions of antigen presentation, co‐stimulation and memory properties of B cells, thus directly or indirectly modulating tumor immunity **(Figure 2)**.^[^ ^26^, ^28^, ^50^, ^70^ ^]^

### 3.1 TIM‐1

TIM‐1, a member of the T cell immunoglobulin and mucin domain‐containing protein family, has been implicated in tumor immunity.^[^ ^26^ ^]^ In gliomas, TIM‐1 expression is significantly elevated in tumor tissues compared to adjacent normal tissues, and this heightened expression is associated with malignant progression and poor patient prognosis.^[^ ^71^ ^]^ A recent study identified TIM‐1 as a specific immune checkpoint on B cells that modulates tumor immune responses.^[^ ^26^ ^]^ TIM‐1 may expressed on all activated B cells and is associated with poor clinical outcomes.^[^ ^26^ ^]^ TIM‐1 can inhibit B cell activation by attenuating the type 1 interferon response of B cells, leading to impaired antigen presentation and co‐stimulatory functions of B cells.^[^ ^26^, ^72^, ^73^ ^]^ Moreover, TIM‐1+ B cells suppress the antitumor effects of CD4+ T and CD8+ T cells while promoting the expansion of FoxP3+ Tregs, thereby indirectly limiting antitumor immunity.^[^ ^26^ ^]^ In addition, flow cytometric analysis revealed that Bregs in HCC tissues highly expresse TIM‐1 protein.^[^ ^49^ ^]^ These B cells mediated immune escape from HCC by secreting IL‐10 through interaction with TIM‐4 expressed on myeloid cells, thereby inhibiting T cell function.^[^ ^49^ ^]^ Interestingly, in another study, knockdown of the gene encoding IL‐10 on TIM‐1+ B cells that highly express IL‐10 did not affect tumor growth.^[^ ^26^ ^]^ This may suggests that TIM‐1's role in promoting tumor escape through alternative mechanisms may greatly outweigh the immunosuppressive effect of IL‐10.

### 3.2 PD‐1/PD‐L1

PD‐1, programmed cell death protein 1, is an immune checkpoint receptor and a member of the immunoglobulin gene superfamily.^[^ ^74^ ^]^ In some tumors, PD‐1 expression has been found to be upregulated in B cells.^[^ ^50^, ^75^ ^]^ PD‐1 is one of the negative regulators of human B cell activation.^[^ ^76^ ^]^ PD‐1 expressed on B cells recruits SHP‐2 to its C‐terminus, leading to the dephosphorylation of key signal transduction molecules for BCR signaling such as Igα/β and SγK, thereby inhibiting BCR signaling.^[^ ^77^ ^]^ In addition, PD‐1 has been shown to affect the memory properties of B cells. PD‐1 expressed by B cells interacts with PD‐L1/PD‐L2 on non‐antigen‐specific cells, thereby inhibiting the generation and reactivation of T cell‐independent memory B cells and negatively regulating antibody production.^[^ ^70^ ^]^ Interestingly, an increased number of memory B cells has been observed in the tumors of patients responding to treatment with PD‐1 inhibitors alone or in combination with CTLA‐4 inhibitors.^[^ ^78^ ^]^ These studies suggest that IC expressed on B cells may contribute to impaired B cell memory function.

High expression of PD‐1 on B cells also impairs the antitumor function of T cells. B cells highly expressing PD‐1 inhibited T cell proliferation and activation by interacting with PD‐L1‐expressing T cells.^[^ ^75^ ^]^ In addition, in a mouse hepatoma model with adoptive transfer of highly PD‐1‐expressing B cells, the infiltration and function of CD8+ T cells were impaired and tumor growth was increased.^[^ ^50^ ^]^ In HCC, a subpopulation of highly PD‐1‐expressing B cells induced IL‐10 secretion through mutual contact with PD‐L1+ monocytes.^[^ ^49^, ^50^ ^]^ IL‐10/IL‐10R signaling drives dysfunction of effector T cells, as evidenced by inhibition of tumor killing by reduced production of interferon‐gamma (IFN‐γ) and cytotoxic granzyme B.^[^ ^50^ ^]^

PD‐L1 is programmed cell death ligand 1.^[^ ^74^ ^]^ Studies have demonstrated that B cells also express PD‐L1 and modulate tumor immunity.^[^ ^79^ ^]^ A subset of tumor‐activated PD‐L1+ B cells promotes the conversion of CD4+ T cell to Tregs through the TGF‐β pathway, which in turn promotes breast cancer progression and metastasis.^[^ ^29^ ^]^ A subsequent study by Zhang et al. using a mouse model of breast cancer also revealed that tumor‐infiltrating B cells (TIBs) highly express PD‐L1.^[^ ^79^ ^]^ These TIBs promote T cell depletion, suppress NK cell proliferation, and inhibit T cell production of cytokines such as IFN‐γ and tumor necrosis factor‐α (TNF‐α), thereby contributing to tumor growth.^[^ ^79^ ^]^ Similar findings were observed in other studies investigating on breast cancer and pancreatic cancer.^[^ ^51^, ^80^ ^]^

### 3.3 CTLA‐4

CTLA‐4, cytotoxic T lymphocyte antigen 4, a member of the immunoglobulin‐related receptors family and participates in immunomodulation upon binding to CD80/CD86.^[^ ^81^ ^]^ Recent studies have demonstrated that CTLA‐4 is expressed in B cells and is associated with tumor progression.^[^ ^27^ ^]^ In a melanoma‐related study, overexpression of CTLA‐4 in B cells was shown to interact with CD86 on antigen‐presenting cells, recruit Tyk2, and activate STAT3 signaling, thereby promoting the expression of immunosuppressive genes and downregulating the anti‐tumor response of Th1 cells.^[^ ^27^ ^]^ Therefore, CTLA‐4 may be one of the ICs regulating the indirect anti‐tumor immune response of B cells.

### 3.4 TIGIT

TIGIT, is an immunosuppressive receptor and a novel IC.^[^ ^82^ ^]^ TIGIT has been found to be expressed on B cells and is associated with tumor progression.^[^ ^28^ ^]^ In gastric cancer patients, a class of TIGIT‐expressing CD20+ B cells was found to infiltrate tumor tissues, and peritumoral CD20+ B cells with high expression of TIGIT was associated with poor prognosis.^[^ ^28^ ^]^ Multiplexed immunofluorescence analyses suggested that these TIGIT‐expressing CD20+ B cells may promote CD8+ T cell depletion and indirectly contribute to the formation of a suppressive TME by upregulating TIGIT expression.^[^ ^28^ ^]^ Furthermore, high TIGIT expression was detected on TIM‐1+ B cells in melanoma tissues and was associated with tumor growth.^[^ ^26^ ^]^

### 3.5 Other Potential B‐Cell ICs

Some B cells express or secrete other immunosuppressive molecules, including inducible T‐cell co‐stimulator ligand (ICOSL), OX40 ligand (OX40L), CD40, and C‐X‐C motif chemokine ligand 13 (CXCL13), which inhibit anti‐tumor immune responses.^[^ ^83^, ^84^, ^85^ ^]^ These immunosuppressive molecules may also function as immune checkpoints for B cells. ICOSL, often interacts with in inducible T‐cell co‐stimulator (ICOS) expressed on T cells to modulate tumor immunity.^[^ ^86^ ^]^ ICOSL expressed on melanoma patient‐derived TNF‐α B cells was found to interact with ICOS on Treg cells and promote FOXP3 + Treg differentiation in a TGF‐β‐dependent manner, which in turn promotes tumor immunosuppression.^[^ ^83^ ^]^ In addition, OX40L, a member of the TNF family, interacts with OX40 to modulate tumor immune responses.^[^ ^87^ ^]^ B cells aberrantly expressing OX40L can bind to OX40 on the surface of T cells and inhibit cytotoxic T lymphocyte (CTL) and IFN‐γ production, thereby limiting anti‐tumor responses.^[^ ^84^ ^]^ As a member of the TNF receptor superfamily, CD40 is a co‐stimulatory molecule expressed by B cells and promotes B cell activation, germinal center (GC) formation, and antibody production upon interaction with CD40L on T cells.^[^ ^88^, ^89^ ^]^ However, CD40 on the surface of B cells can interact with CD40L expressed by tumor cells to promote B cell secretion of IL‐10, which is negatively correlated with IFN‐γ production by CD8+ T cells and NK cells.^[^ ^85^ ^]^ Furthermore, it has been proposed that CXCL13 is an immune checkpoint for Bregs in the metastatic microenvironment of tumors.^[^ ^90^ ^]^ This study found that Bregs expressing C‐X‐C motif chemokine ligand 5 (CXCL5) were reduced when CXCL13 was defective, as well as Bregs recruited to the tumor microenvironment.^[^ ^90^ ^]^ CXCL13 can be secreted by cancer‐associated fibroblasts (CAFs) and cancer cells.^[^ ^91^ ^]^ This suggests that tumor cells may promote tumor progression by regulating B cell function through CXCL13 and CXCL5 (**Figure** **2**).

## 4 Comparison of B‐Cell ICs and T‐Cell ICs

**Figure 3:**
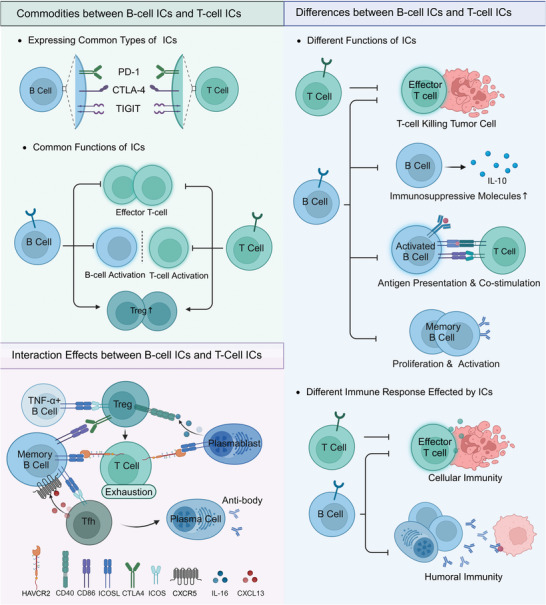
Comparison of B‐cell ICs and T‐cell ICs. B‐cell ICs and T‐cell ICs share similarities in that they are the same type of ICs and exhibit comparable actions. However, B‐cell ICs and T‐cell ICs differ in their specific functions and mechanisms of action. T‐cell ICs primarily influence the antitumor immune response mediated by T cells, while B‐cell ICs can additionally impact various B cell functions, such as the secretion of immunosuppressive molecules, antigen presentation, co‐stimulation, and the proliferation and activation of memory B cells. Consequently, T‐cell ICs are primarily involved in antitumor cellular immunity, while B‐cell ICs can also modulate humoral immunity. Furthermore, interactions may occur between IC ligands and receptors of different subpopulations of B cells and T cells. Abbreviations: IC, immune checkpoint; Treg, regulatory T cell; Tfh, T follicular helper cell. This figure was created based on the tools provided by Biorender.com.

T‐cell ICs in tumors have been widely studied in the last two decades or so. Based on the close connection and molecular mechanism between B‐cell ICs and tumor immunity, we summarize and compare the roles of B‐cell ICs and T‐cell ICs. During tumorigenesis and progression, some ICs whose expression is upregulated on T cells are also upregulated on B cells.^[^ ^27^, ^49^, ^50^ ^]^ There are similarities and differences in the roles of these same IC molecules on T and B cells. In addition, there are differences in the roles and their mechanisms performed by different ICs of B cells and T cells in tumor immunity (**Figure** **3**).

### 4.1 Commonalities between B‐Cell ICs and T‐Cell ICs

In tumors, IC molecules that are aberrantly upregulated on B cells partially overlap with IC molecules expressed on T cells. T‐cell‐expressed ICs associated with tumors, including PD‐1, CTLA‐4, and TIGIT, are also aberrantly upregulated in B cells and are associated with pro‐tumor immunity.^[^ ^27^, ^28^, ^50^, ^92^, ^93^, ^94^ ^]^ In addition, the same class of IC signals expressed by both B cells and T cells can influence the antitumor effects of T cells. As described above, B cells with upregulated PD‐1 or TIGIT expression promote CD8+ T cell depletion, an effect that is also present in T cell IC.^[^ ^28^, ^95^ ^]^) For example, in vitro tumor cell line experiments have shown that PD‐1 expression on CTLs also has a role in negatively regulating tumor killing by CTLs themselves.^[^ ^50^, ^96^ ^]^ Additionally, a transcriptome analysis revealed that in hepatocellular carcinoma, TIGIT‐expressing tumor‐infiltrating CD8+ T cells are often depleted, thus limiting the effector function of T cells.^[^ ^97^ ^]^

Different IC molecules upregulated in T and B cells also exhibit the same immune effects. Upregulated expression of ICs negatively regulates the activation of the cells themselves and affects antitumor immunity. PD‐1 on the surface of T cells directly regulates T‐cell receptor (TCR) signaling to attenuate T‐cell activity.^[^ ^98^ ^]^ T‐cell‐expressed IC molecules, such as CTLA‐4 and BTLA, also inhibit T‐cell activation.^[^ ^92^, ^99^ ^]^ In B cells, upregulation of TIM‐1 hinders B cell activation and limits the involvement of B cells in the antitumor immune response.^[^ ^26^ ^]^ In addition, through activation of IC signaling, both T cells and B cells can affect the function of Tregs in the TME. An experimental animal study found an increase in Tregs expressing TIGIT in ovarian cancer, which mediates the immunosuppressive function of Tregs.^[^ ^100^ ^]^ The PD‐L1 molecule expressed on B cells also promotes the transformation of CD4+ T cells into Tregs.^[^ ^29^ ^]^ ICOSL on the surface of B cells also binds to ICOS on Tregs to promote Treg proliferation.^[^ ^83^ ^]^ All of these ICs indirectly inhibit antitumor immunity by promoting the effects of Tregs.

### 4.2 Differences between B‐Cell ICs and T‐Cell ICs

Current evidence suggests that the primary pathways involved in tumor immunity differ between T‐cell ICs and B‐cell ICs. T‐cell ICs primarily exhaust T‐cells and inhibit their cytotoxic effects in order to directly impede the antitumor immune response.^[^ ^50^, ^93^, ^94^, ^101^ ^]^ For example, studies have demonstrated that tumor‐infiltrating CD8+ T cells with high TIGIT expression can limit the effector function of these cells.^[^ ^101^, ^102^ ^]^ In a study analyzing specimens from pancreatic cancer patients, an increase in T‐cell TIGIT expression within the tumor was identified, which correlated with a T‐cell depletion phenotype.^[^ ^94^ ^]^

Unlike T‐cell IC, which directly depletes itself, B‐cell ICs primarily influence T‐cell function and secretion of immunosuppressive molecules, indirectly suppressing antitumor immunity.^[^ ^49^, ^50^, ^85^ ^]^ Bregs with high expression of TIM‐1 not only secreted large amounts of IL‐10, but also inhibited the function of CD8+ T cells, thereby attenuating anti‐tumor immunity.^[^ ^49^ ^]^ Similar effects were observed in Breg with high expression of PD‐1.^[^ ^50^ ^]^ Furthermore, B‐cell ICs can significantly impact B‐cell function. The TIM‐1 checkpoint molecule expressed on B cells inhibits their antigen presentation and co‐stimulatory.^[^ ^26^ ^]^ PD‐1 expressed by B cells may impair their memory properties.^[^ ^70^ ^]^ Impairment of these functions in B cells often affects antitumor immunity. The effect of IC expressed by T cells on the anti‐tumor function of B cells is currently unknown.

There are also differences in the immune responses affected by B‐cell ICs compared to T‐cell ICs. T cell ICs often hinders the cellular immune response in TME due to their functions in depleting T cells and limiting T cell cytotoxicity.^[^ ^92^, ^94^, ^101^ ^]^ Activated B cells have been demonstrated to produce tumor‐specific antibodies that exert anti‐tumor effects.^[^ ^6^, ^63^ ^]^ High expression of IC by B cells, such as TIM‐1, may limit B cell activation.^[^ ^26^ ^]^ Thus, the presence of B cell IC signaling may have a detrimental effect on antibody production by B cells. Therefore, in addition to limiting cellular immunity, B‐cell ICs may also affect humoral immune responses involving tumor‐specific antibodies, potentially further amplifying the immunosuppressive signals.^[^ ^26^ ^]^

### 4.3 Interaction Effects between B‐Cell ICs and T‐Cell ICs

There may be crosstalk between B‐cell ICs and T‐cell ICs in the TME. Single‐cell sequencing identified pathways for T‐cell‐B‐cell interactions in triple‐negative breast cancer.^[^ ^103^ ^]^ Plasmablasts and memory B cells chemotaxis Tregs into the TME via the IL‐16‐CD40 and CD86‐CTLA‐4 pathways, respectively. LGALS9 on the surface of memory B cells also exhausts CD8+ T cells by binding to HAVCR2 on CD8+ T cells. In addition, B cells and T cells are involved in suppressive TME through the ICOSLG‐CTLA4 and CD86‐CTLA4 pathways. Interestingly, B cell‐T cell interactions also provide favorable conditions for antitumor immunity. Memory B cells and T cells through ICOSLG‐ICOS signaling can promote the differentiation of B cells into antibody‐producing plasma cells in the GC. Memory B cells expressing CXCR5 also attract CXCL13‐secreting T follicular helper (Tfh) cells, driving plasma cell differentiation. However, a previous study found that the co‐stimulatory ligand ICOSL expressed on TNF‐α B cells is pro‐tumorigenic after interacting with ICOS on Tregs.^[^ ^83^ ^]^ This stems in part from the complex roles of different B cell subsets in the TME. In conclusion, B cells and T cells can cooperate to exert pro‐tumorigenic effects through signal exchange of ICs.

## 5 Targeting B‐Cell IC Contributes to the Restoration of Anti‐Tumor Immunity

**Figure 4:**
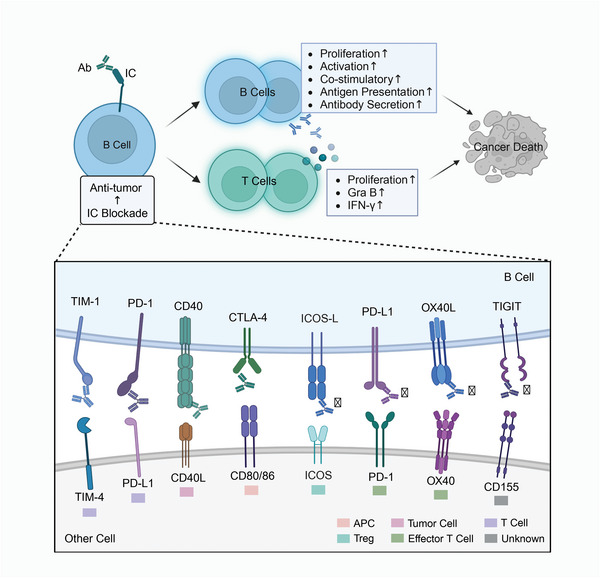
B‐cell immune checkpoint blockade restores anti‐tumor immunity. Blockade of aberrant IC signaling on B cells may facilitate the restoration of B cell function while relieving the inhibitory effect of B cells on T cell effector function. The use of antibodies targeting IC molecules restores B‐cell proliferation, activation, antigen presentation, and co‐stimulatory functions, and may contribute to the activation of humoral immunity by B‐cell production of tumor‐specific antibodies. In addition, the anti‐tumor effector function of T cells is restored, as evidenced by increased T cell infiltration and production of more granzyme B and IFN‐γ, and enhanced tumor killing. Abbreviations: IC, immune checkpoint; IFN, interferon; APC, antigen‐presenting cell; MDSC, myeloid‐derived suppressor cell; Treg, regulatory T cell; Gra B, Granzyme B. This figure was created based on the tools provided by Biorender.com.

Blocking abnormal IC signals on B cells can reverse dysfunctional B cells and restore the anti‐tumor effects of B cells (**Figure** **4**).^[^ ^26^, ^52^ ^]^ Currently, there are very few studies exploring the tumor immune response and its molecular mechanisms after ICI blockade of B‐cell IC. Here, we reviewed as many studies as possible on ICI blockade associated with B‐cell IC to explore the potential mechanisms associated with targeting B‐cell IC to restore anti‐tumor immunity. From current evidences, ICIs may restore the proliferation, activation, antigen presentation, and co‐stimulation of B cells, and relieve the inhibition of T cell function, assisting and amplifying the tumor‐killing effects of T cells; promote the production of B‐cell antibodies and activate the humoral immune response, inducing more powerful anti‐tumor immunity.^[^ ^26^, ^27^, ^85^, ^104^ ^]^ This suggests that B‐cell ICs may be a new target for tumor immunotherapy.

### 5.1 ICIs Restores Proliferation, Activation, Antigen Presentation, and Co‐Stimulation of B Cells

Blocking aberrant IC signals of TIM‐1, PD‐1, and CTLA‐4 on B cells may help restore B cell functions, including proliferation, activation, antigen presentation, and co‐stimulation. In a mouse model of melanoma, knockdown of the gene encoding TIM‐1 in B cells or treatment with an anti‐TIM‐1 antibody resulted in stronger expression of the type I interferon‐responsive gene signature in B cells. This lowered the BCR activation threshold, thereby enhancing the B cell activation response and ability to present antigen to CD4+ T cells, contributing to the role of B cells in tumor immunity.^[^ ^26^ ^]^ Inhibition of CTLA‐4 promotes B cell activation and expansion, which is a prerequisite for B cells to fight tumor antigens.^[^ ^105^ ^]^ In a study by Jo et al., increased activation and infiltration of a large number of tumor‐reactive B cells were observed, accompanied by a reduction in tumor volume, in a syngeneic mouse tumor transplant model treated with a CTLA‐4 antibody.^[^ ^105^ ^]^ PD‐1 monoclonal antibody‐treated patients with esophageal squamous carcinoma had elevated levels of naïve B cells and plasma cells and were associated with longer PFS and OS.^[^ ^106^ ^]^ This suggests that proliferative activation of B cells may be positively regulated after blocking PD‐1. In a previous study by Thibult et al, the proliferation and activation of B cells was significantly enhanced when PD‐1 blocking antibodies were utilized against PD‐1 on B cells.^[^ ^76^ ^]^

### 5.2 ICIs Enhance Antibody Secretion and Activate Humoral Immune Response of B Cells

Blockade of aberrant IC signaling on the surface of B cells may enhance antibody secretion by B cells as well as induce humoral immune responses for antitumor effects. Tumor‐specific antibodies secreted by B cells are thought to be part of the tumor immune microenvironment and participate in the tumor immune response.^[^ ^107^ ^]^ Depletion of B cells promotes the growth of HPV‐associated squamous cell carcinomas, suggesting that B cells play a critical role in inhibiting tumor progression.^[^ ^108^ ^]^ In an non‐small cell lung cancer (NSCLC) cohort with the treatment of a PD‐L1 antibody, elevated levels of B cells and plasma cells were found to be associated with improved clinical outcomes.^[^ ^109^ ^]^ Using single‐cell RNA sequencing and BCR sequencing analysis, Kim et al. found that in an animal model of HPV‐associated squamous cell carcinoma, anti‐PD‐L1 antibody treatment promoted the maturation and differentiation of B cells into memory B cells, plasma cells, and antigen‐specific B cells, and consequently induced GC formation.^[^ ^108^ ^]^ Subsequently, Kim et al. conducted a phase II randomized clinical trial and observed an increase in IgG and IgM antibody production in the sera of patients treated with PD‐1 blockade, further demonstrating that immune checkpoint blockade (ICB) promotes the activation of humoral immunity mediated by B cells.^[^ ^108^ ^]^ In addition, treatment with anti‐CTLA‐4 antibody activates the humoral immune response, inducing the production of antitumor‐specific antibodies.^[^ ^105^ ^]^ A study related to melanoma showed that treatment with an anti‐CTLA‐4 antibody significantly inhibited tumor growth by deregulating antitumor immunity through inhibition of CTLA4‐STAT3 signaling in B cells.^[^ ^27^ ^]^ However, anti‐TIM‐1 antibody treatment did not significantly affect B cell antibody secretion and humoral immune responses.^[^ ^110^ ^]^

### 5.3 ICIs Release the Inhibitory Effect of B Cells on T Cell Anti‐Tumor Immunity

B‐cell IC signaling affects the function of other immune cells in the TME, and blocking B‐cell ICs deregulates this abnormality and restores the immune response effect of these cells, which is mainly manifested in the restoration of the anti‐tumor effect of effector T cells. For example, treatment of melanoma mice with TIM‐1 antibody resulted in increased infiltration of CD4 and CD8 T cells as well as increased production of granzyme and IFN‐γ by T cells.^[^ ^26^ ^]^ In an animal model of breast cancer, use of PD‐1 monoclonal antibody to block the PD1 – PD‐L1 interactions between B cells and tumor‐derived MDSCs attenuated the PD1+ B‐cells’ inhibitory effects on T‐cell proliferation and IFN‐γ secretion, as evidenced by reduced tumor growth.^[^ ^51^, ^52^ ^]^ Thus, blocking B cell Ics restores the role of B cells in assisting T cells to exert antitumor toxic effects.

### 5.4 Association of B‐Cell ICs with TLS

The TLS of the TME is one of the B‐cell settlements and there may be a link with B‐cell ICs. TLS is observed in the TME and is one of the predictors of ICI response efficiency.^[^ ^111^ ^]^ The GC is a major part of the mature TLS and is one of the sites of B‐cell affinity for maturation and class switching, which contributes to effective humoral immune response.^[^ ^12^, ^112^ ^]^ An increase in GC B cells in TLS has been associated with improved clinical outcomes in tumor patients.^[^ ^113^ ^]^ It has been proposed that one of the reasons for the enhanced B cell response to ICI may be that PD‐1/PD‐L1 antibodies simultaneously deregulate the inhibitory effect of PD‐1/PD‐L1‐expressing B cells on tumor immunity.^[^ ^114^ ^]^ B‐T cell synergy may also be one of the mechanisms of the favorable prognostic effect of TLS B cells on ICI therapeutic response.^[^ ^115^ ^]^ CAFs, which have histiocyte characteristics of lymphoid tissues, play a coordinating role in the formation of TLS in murine melanoma. In turn, CAF accumulation and TLS expansion are dependent on CXCL13‐mediated recruitment of B cells expressing lymphotoxin α1β2.^[^ ^116^ ^]^ An experimental study in animals with triple‐negative breast cancer demonstrated that the activation of B‐cell‐activated Tfh cells, T‐cell‐induced B‐cell activation, and antibody production play an important role in ICI therapeutic response.^[^ ^117^ ^]^ In addition, during anti‐PD‐1 therapy, tumor TLS B cell activation and increased antibody production were found to be possibly associated with an increase in peripheral blood cTfh.^[^ ^118^ ^]^ Thus, B cell ICs may influence TLS function by complex mechanisms.

## 6 B‐Cell Response and ICI Activity in Solid Tumors

B cells express PD‐1 and CTLA‐4, and it is theorized that the immune response of B cells would be affected by treatment with PD‐1 and CTLA‐4 inhibitors. Recent studies have demonstrated that B‐cell responses correlate with the activity of ICIs in the treatment of solid tumors^[^ ^119^, ^120^ ^]^ (**Table** **1**), although there is currently no direct evidence that these ICIs target B‐cell‐expressed ICs.

**Table 1:** B cell response associated with ICI treatment in solid tumors.

Response of B‐cell	Type of B‐cell	Treatment	Tumor	Clinical Outcomes
High infiltration	B cell	Anti CTLA‐4	Melanoma	Longer OS^[^ ^121^ ^]^
	B cell	Anti PD‐1	HNSCC	Longer OS^[^ ^120^ ^]^
	B cell	Anti PD‐1	Soft‐tissue sarcomas	Higher response rate Longer OS^[^ ^111^ ^]^
	CD19 B cell	Anti PD‐L1+taxane	TNBC	Higher response rate^[^ ^122^ ^]^
	Memory B cell	Anti PD‐1	RCC	Higher response rate Longer OS and PFS^[^ ^123^ ^]^
	Memory B cell	Anti PD‐1	NSCLC	Longer PFS^[^ ^124^ ^]^
	Plasma cell	Anti PD‐1	NSCLC	Higher response rate Longer OS^[^ ^109^ ^]^
	Plasmablast & naive‐like B cell	Anti PD‐1/Anti PD‐1+anti CTLA‐4	Melanoma	Higher response rate^[^ ^32^ ^]^
High gene expression signature	B cell	Anti PD‐1	Soft‐tissue sarcomas	Higher response rate Longer OS^[^ ^111^ ^]^
	B cell	Anti PD‐1	Lung adenocarcinoma	Longer PFS^[^ ^126^ ^]^
	B cell	Anti PD‐1/Anti PD‐1+ Anti CTLA4	RCC	Higher response rate^[^ ^78^ ^]^
	B cell	Anti PD‐1/Anti CTLA4 /Anti PD‐1+ Anti CTLA4	Melanoma	Higher response rate^[^ ^78^ ^]^
	B cell	anti‐PD1 with bevacizumab and oral cyclophosphamide	Ovarian cancer	Higher response rate Longer PFS^[^ ^125^ ^]^
	Memory B cell	Anti PD‐1	Melanoma, Urothelial carcinoma	Higher response rate Longer PFS^[^ ^127^ ^]^
	Plasmablast	Anti PD‐1	Melanoma	Longer OS^[^ ^32^ ^]^
	Plasma cell	Anti PD‐1	NSCLC	Longer OS^[^ ^109^ ^]^
BCR diversity &clonal expansion	B cell	Anti PD‐1/Anti CTLA4 /Anti PD‐1+ Anti CTLA4	Melanoma	Higher response rate^[^ ^78^ ^]^
BCR signature	B cell	anti‐PD1 and/or anti‐CTLA4	Melanoma	Higher response rate Longer OS^[^ ^128^ ^]^
BCR diversity	Plasmablast	Anti PD‐1/Anti PD‐1+anti CTLA‐4	Melanoma	Higher response rate^[^ ^105^ ^]^
TLS		Anti CTLA‐4	Melanoma	Longer OS^[^ ^121^ ^]^
		Anti PD‐1	NSCLC	Longer OS^[^ ^109^ ^]^
		Anti PD‐1	Soft‐tissue sarcomas	Higher response rate Longer OS^[^ ^111^ ^]^

Abbreviations: OS, overall survival; PFS, progression‐free survival; HNSCC, head and neck squamous cell carcinoma; TNBC, triple‐negative breast cancer; RCC, renal cell carcinoma; NSCLC, non‐small cell lung cancer; BCR, B cell receptor; TLS, tertiary lymphoid structure.

### 6.1 B‐Cell Infiltration

In patients with tumors treated with CTLA‐4 or PD‐1 inhibitors, B‐cell infiltration is associated with the immune response and clinical prognosis. In the treatment of melanoma, HNSCC and soft‐tissue sarcomas with ICIs, high B‐cell infiltration was associated with longer overall survival (OS) in patients.^[^ ^111^, ^120^, ^121^ ^]^ A single‐cell sequencing analysis found that triple‐negative breast cancer (TNBC) responders treated with atezolizumab in combination with taxane had an abundance of CD19 B cells.^[^ ^122^ ^]^ In addition, infiltration of memory B cells was observed in responders with RCC treated with nivolumab and was associated with prolonged OS and progression‐free survival (PFS).^[^ ^123^ ^]^ In anti‐PD‐1 antibody‐treated NSCLC, an increase in memory B cells was associated with longer PFS,^[^ ^124^ ^]^ and an increase in plasma cells was associated with a higher response rate and longer OS.^[^ ^109^ ^]^ The correlation between plasmablasts and naive‐like B cells with a high frequency of ICI response was confirmed in the melanoma cohort.^[^ ^32^ ^]^

### 6.2 Gene Signatures Related to B‐Cells

Several studies have demonstrated the relationship between B‐cell‐related features and ICI activity. The correlation between B‐cell gene expression signatures and ICI response rates has been characterized in a variety of tumors, including soft‐tissue sarcomas, RCC, melanoma, and ovarian cancer.^[^ ^78^, ^111^, ^125^ ^]^ In addition, in lung adenocarcinoma and ovarian cancer, B‐cell profiles predicted longer PFS after ICI treatment.^[^ ^125^, ^126^ ^]^ A study by Varn et al. revealed that memory B‐cell‐derived gene profiles were correlated with higher response rates and longer PFS in melanoma and urothelial carcinoma patients treated with ICIs.^[^ ^127^ ^]^ Improved OS in melanoma patients treated with PD‐1 inhibitors was also significantly correlated with plasmablast characterization.^[^ ^32^ ^]^

### 6.3 BCR

The effect of BCR on ICI activity has primarily been investigated in melanoma cohort‐related studies. BCR diversity and clonal expansion were predictive of response in melanoma patients treated with PD‐1 inhibitor or CTLA‐4 inhibitor or a combination of PD‐1 inhibitor and CTLA‐4 inhibitor.^[^ ^78^ ^]^ Furthermore, an additional study showed that improved ICI response activity in melanoma was also correlated with the BCR diversity of plasmablasts.^[^ ^105^ ^]^ A study by Arian et al reported that BCR signature predicted response rate and OS in melanoma patients treated with a PD‐1 inhibitor or a combination of PD‐1 and CTLA‐4 inhibitors.^[^ ^128^ ^]^

### 6.4 TLS

TLS is also a determinant of ICI response. In melanoma, the presence of mature TLS has been shown to be significantly associated with increased OS following ICI treatment.^[^ ^121^ ^]^ Similar results have been demonstrated in studies investigating NSCLC and soft‐tissue sarcomas.^[^ ^109^, ^111^ ^]^

## 7 Conclusion and Future Perspectives

Tumor immunotherapy has emerged as a major breakthrough in the field of tumor treatment due to its ability to elicit durable and sustained responses.^[^ ^17^, ^18^ ^]^ ICI is one of the most representative tumor immunotherapy modalities. classical ICB therapies targeting T‐cell‐expressed ICs such as such as PD‐1 and CTLA‐4, have become the standard of care for various tumors, leading to significant improvements in patient survival in clinical settings.^[^ ^129^, ^130^, ^131^ ^]^ For decades, T cells have been the central focus of ICB development. In recent years, the increasing recognition that B cells can generate tumor‐specific antibodies and mount anti‐tumor humoral immunity has underscored the crucial role of B cells in the anti‐tumor immune response.^[^ ^132^, ^133^ ^]^ The discovery of ICs expressed on B cells undoubtedly marks the beginning of a new era in B‐cell IC‐based immunotherapy. In addition to the recent discovery of TIM‐1, earlier studies have identified IC molecules, such as PD‐1 and CTLA‐4 that are expressed on B cells and are involved in tumor immune escape.^[^ ^26^, ^27^, ^29^, ^30^ ^]^ Targeting B‐cell ICs restores the anti‐tumor function of B cells and potentiates the tumoricidal activity of T cells. Therefore, it is crucial to cultivate the potential of B‐cell ICs in tumor therapy, as this will likely provide cancer patients with expanded therapeutic options, improved efficacy, and better clinical outcomes. Currently, the relationship between B cell‐expressed ICs and anti‐tumor immunity remains in its infancy, with numerous questions deserving further investigation and resolution.

### 7.1 Is There a Link between B‐Cell Exhaustion and B‐Cell ICs?

Lymphocyte depletion is one of the hallmarks of the immunosuppressive TME and may be associated with high expression of ICs.^[^ ^134^, ^135^ ^]^ Abnormal IC signaling is involved in T‐cell depletion.^[^ ^134^ ^]^ In T‐cells, upregulation of PD‐1 and CTLA‐4 often causes T‐cell depletion, which manifests as impaired immune response of the T‐cells and thus promotes tumor progression.^[^ ^136^ ^]^ B‐cell exhaustion is also present in the TME.^[^ ^137^ ^]^ Researchers have identified exhausted tumor‐infiltrating lymphocyte B‐cells (TIL‐B cells) (CD69+HLA‐DR+CD21‐CD27‐) in patients with NSCLC.^[^ ^137^ ^]^ The antigen‐presenting function of these TIL‐B cells remains normal, but they are associated with an increase in Tregs, suggesting that exhausted B cells may suppress tumor immunity by promoting the conversion of effector T cells into Tregs. However, there is still a gap regarding the link between B cell exhaustion and B‐cell ICs. In tumors, do B cell ICs lead to B cell exhaustion? Or is there an aberrant expression of tumor‐associated ICs in exhausted B cells? In the future, it is important to identify more exhausted B‐cells in tumors using single‐cell sequencing technology, which is beneficial to explore whether there is a link between B‐cell ICs and the status and function of exhausted B‐cells.^[^ ^138^ ^]^

### 7.2 Is There Spatiotemporal Heterogeneity of B‐Cell ICs in Tumors?

Based on the current findings, tumor‐immunity‐related ICs in B‐cells have been identified in specific B‐cell subpopulations. With the exception of TIM‐1, there is no evidence yet that these ICs are expressed abnormally in all B cells. Given the existence of different surface markers, complex subpopulations of B cells, and differences in their functions,^[^ ^139^ ^]^ we ask the question whether there is spatiotemporal heterogeneity in tumor‐associated B‐cell ICs, i.e., whether the expression and roles of B cell ICs show heterogeneity in different B cell subpopulations and developmental stages, as well as in different tumor types and infiltration sites.

Is there heterogeneity in the expression of tumor ICs in different developmental lineages and subpopulations of B cells? TIGIT seems to be overexpressed in memory B cells and CD20+ B cells,^[^ ^28^ ^]^ CXCL13 is an IC in Bregs,^[^ ^90^ ^]^ and ICOSL is overexpressed in TNF‐α‐expressing B cells,^[^ ^83^ ^]^ suggesting that the types of B‐cell ICs may vary according to the subpopulations of B cells. In addition to B‐cell subpopulations, are there changes in the type, level and function of tumor‐regulated B‐cell IC expression when B cells are at different developmental stages? What are the regulatory mechanisms of such changes? These need to be further explored. Since the function of B cells varies by subpopulation,^[^ ^139^ ^]^ the study and understanding of the heterogeneity of B‐cell ICs is necessary and important.

Is there heterogeneity in the expression of tumor‐associated B‐cell ICs in tumor types and B‐cell infiltration sites? Currently, melanoma animal models are usually chosen for the identification of B‐cell ICs such as TIM‐1, CXCL13, and CTLA‐4, and the effects of these ICs present positive results only in melanoma.^[^ ^26^, ^27^, ^83^ ^]^ Can B‐cell ICs in other tumors exhibit similar functions to those presented in melanoma? In addition, because of the possible heterogeneity of TME, tumor cells, and B‐cell infiltration sites in different cancers,^[^ ^140^ ^]^ it is not clear whether there are differences in the expression and function of B‐cell ICs due to these heterogeneities. For example, CXCL13 has been suggested to be a novel immune checkpoint for Bregs.^[^ ^90^ ^]^ However, CXCL13 is one of the markers of TLS formation.^[^ ^141^ ^]^ Is this contradictory conclusion caused by differences in the expression of ICs and their roles at different B‐cell infiltration sites?

### 7.3 Does ICIs Directly Affect B Cell Activation and Function?

B cells are functionally active during ICI treatment of tumors. During melanoma treatment, plasma cells and IgG antibodies were increased in the peripheral blood of responders to PD‐1 inhibitors, CTLA‐4 inhibitors, or a combination of PD‐1 inhibitors with CTLA‐4 inhibitors compared with nonresponders.^[^ ^142^, ^143^, ^144^ ^]^ The increase in IgG antibodies in these responders was associated with a better tumor prognosis,^[^ ^143^ ^]^ suggesting that IgG antibodies secreted by B cells may play a role in the ICI antitumor process. Increased numbers of B cells and memory B cells were also found in tumors of responders to PD‐1 inhibitors or combination PD‐1 inhibitors with CTLA‐4 inhibitors.^[^ ^78^ ^]^

It is unclear whether PD‐1 inhibitors and CTLA‐4 inhibitors have a direct effect on B cells. In the TME, T cells upregulate the expression of PD‐1 and CTLA‐4, which promotes tumor immune escape.^[^ ^18^, ^24^, ^25^ ^]^ PD‐1 and CTLA‐4 have also been found to be upregulated in expression on B cells and correlated with tumor immunity.^[^ ^27^, ^50^, ^75^ ^]^ T‐cell‐based ICI restores the antitumor function of T cells mainly by inhibiting the expression of ICs on T cells.^[^ ^25^, ^145^ ^]^ Therefore, it remains to be further investigated regarding the specific link between the antitumor effects of B cells, functionally restored T cells, and T‐cell ICIs. Future questions to be addressed include: 1) Are the same immune checkpoints on B cells as on T cells also inhibited by T cell ICI? 2) Are the elevated levels of B cells and IgG antibodies in PD‐1 inhibitor and/or CTLA‐4 inhibitor responders due to direct blockade of B cell ICs and promotion of B cell antitumor effects, or are they indirectly promoting B cell activation by restoring T cell function through blockade of T‐cell ICs?

### 7.4 Potential Strategies for Exploiting B‐Cell ICs in Tumors

The discovery of B‐cell tumor ICs indicates a new direction in the field of tumor immunology research.^[^ ^26^ ^]^ However, B‐cell tumor‐associated ICs have not been extensively studied at present. It is to be expected that more B‐cell ICs in tumors will be identified in the future. Summarizing and learning from previous tumor IC development techniques can help to advance this process.

The identification of the B‐cell‐specific tumor IC TIM‐1 lays the foundation for subsequent mining of B‐cell ICs. Researchers identified a specific class of B‐cell subpopulation using single‐cell RNA sequencing (scRNA‐seq) in a melanoma mouse model and characterized the subpopulation‐specific marker (potential IC), TIM‐1.^[^ ^26^ ^]^ Subsequently, the researchers observed the effect of TIM‐1 expression on tumor growth through elimination of the functionality of potential ICs with deletion of the Havcr1 gene or using TIM‐1 antibody. Afterward, the researchers further explored the mechanisms underlying the link between the two. This included testing the infiltration of lymphocytes, the antigen‐presenting function of B cells, and the tumor‐killing function of T cells. This study lays the theoretical knowledge of experimental techniques for future B cell IC discovery. Given the complexity of B‐cell subpopulations and their functional relevance, the identification of B‐cell subpopulations and their surface markers may be one of the breakthroughs for further exploration of tumor ICs on B‐cells. B‐cell subpopulations are often identified with the help of scRNA‐seq technology;^[^ ^146^ ^]^ however, this technology is still an obstacle to the typing of B‐cell subpopulations, especially in the identification of Breg cell subpopulations.^[^ ^138^ ^]^ Therefore, scRNA‐seq technology needs to be further optimized in the future.

The discovery of T‐cell ICs in tumors provides a referable research idea for the identification of B‐cell ICs. Here we focus on reviewing the discovery process of several ICs in T cells, including PTP1B, CD96, Siglec‐5, and PCBP1. Potential ICs can start from a certain kind of immune‐associated molecule known to be expressed in T cells or ICs expressed in other immune cells. For example, previous studies have identified CD96 as an IC for NK cells;^[^ ^147^ ^]^ Siglec‐5 is one of the key molecules for innate immune action.^[^ ^148^ ^]^ When identifying Siglec‐5 and PCBP1, researchers started by investigating the role of the ICs in other models of disease, and later explored and validated the ICs in an animal tumor model.^[^ ^149^, ^150^ ^]^ Monitoring the potential IC expression on T cells is also important, as this often suggests a tumor association. Abnormal upregulation of PTP1B, CD96, and Siglec‐5 correlates with tumor immunosuppression, whereas upregulation of PCBP1 favors antitumor immunity.^[^ ^149^, ^150^, ^151^, ^152^, ^153^ ^]^ Tumor growth was observed and relevant mechanisms were explored after blocking potential IC using gene deletion or monoclonal antibodies. For example, during the identification of PTP1B ICs, researchers observed whether the number, activation, and function of PTP1B‐deficient T cells were affected.^[^ ^151^ ^]^ Expression of PTP1B, CD96, and Siglec‐5 was also examined in human cancer tissues,^[^ ^150^, ^151^, ^152^, ^153^ ^]^ which may help to attenuate the differences between animal models of tumors and human cell lines. Finally, the function and mechanism of the potential ICs was verified by targeting this IC in tumor models. For example, systemic inhibition of PTP1B with small molecules was observed to enhance anti‐tumor immunity.

**Figure 5:**
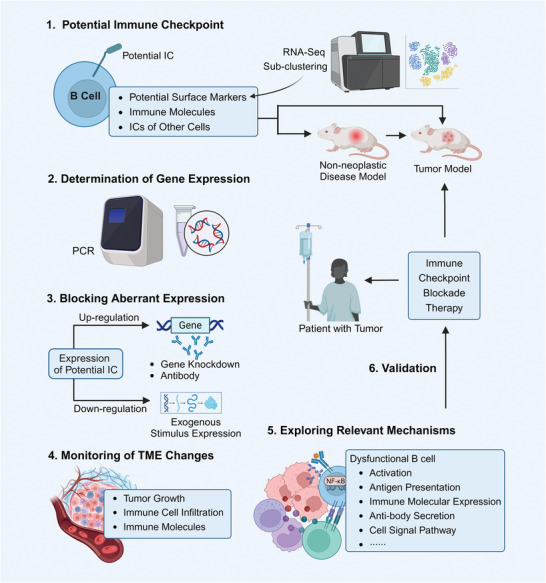
Potential strategies for B‐cell IC development. Starting from the identified tumor ICs of other immune cells, immune molecules, or surface markers abnormally expressed by B cells, the potential IC molecules of B cells are initially identified. The expression of this IC is determined using PT‐PCR. Afterward, the abnormal expression of this IC is blocked, and changes in tumor growth and immune infiltration are observed. Revealing the mechanism of the role of B‐cell IC in tumor immunity also requires further exploration of the effects of IC on B‐cell function and other immune cell functions. Finally, after obtaining relevant positive results in tumor animal models, the role of this IC in tumor progression is verified in clinical cohorts using relevant ICI. Abbreviations: IC, immune checkpoint. This figure was created based on the tools provided by Biorender.com.

Therefore, based on the above referable approaches and theories, we propose potential strategies for B‐cell tumor IC identification (**Figure** **5**): 1) Screening of the potential tumor IC can be based on the ICs of other immune cells, tumor immune‐related molecules abnormally expressed by B cells, and identified B‐cell surface markers. 2) The expression of the potential tumor IC on the B‐cell surface is usually reflected by mRNA expression measured by PT‐PCR. 3) Using tumor animal models, gene knockdown, monoclonal antibodies or activating protein expression was selected to block the function of the potential IC based on the abnormal expression (up‐regulation or down‐regulation), to further observe tumor growth and the infiltration of lymphocytes, including T cells and B cells. 4) Prior to this process it is also desirable to study the role of the potential IC in other diseases.5) Once it has been determined that the potential IC has a potential effect on tumor immunity, it is important to explore whether B‐cell activation, B‐cell function, T‐cells, and other immune cells are affected by detecting immune cell numbers, immune molecule levels, antibody levels, and signaling pathways, in order to reveal the mechanism of the potential IC in tumor immunity. 6) Since there may be some differences between animal tumor models and human tumors, it is necessary to validate the role of the potential IC in human tumors. Use human tumor cell lines to detect the expression of B‐cell potential IC, tumor growth, etc., as well as to explore the effect of blocking the potential IC. The role of B‐cells in tumor immunity has just been unveiled. The identification of B‐cell subpopulations and their surface markers may lay the foundation for B‐cell tumor IC studies. Therefore, identification of more B cell surface markers in tumors is one of the future research directions.

### 7.5 Targeting B‐Cells in Combination with Other Immunotherapies is Expected to be a New Direction in Tumor Treatment

The combination of multiple ICIs improves the clinical dilemma of poor efficacy of single ICI. Studies have shown that the combination of multiple ICIs is beneficial for improving the efficacy of tumor therapy compared with single ICI therapy.^[^ ^154^ ^]^

As the role of B cells in tumor immunity continues to be revealed, therapeutic modalities targeting B cells are undoubtedly one of the candidates for future tumor therapy. In a mouse model of triple‐negative breast cancer, B cells enhanced the antitumor immune effects of ICI in the form of antibody secretion and helper T‐cell responses.^[^ ^117^ ^]^ In addition, B‐cell‐expressed PD‐1 inhibits T‐cell activation and thus is detrimental to the efficacy of ICI.^[^ ^75^ ^]^ Therefore, targeting B‐cell IC in combination with T‐cell ICI may help improve ICI efficacy. Due to differences in anti‐tumor and pro‐tumor effects of different B‐cell subsets, depletion of specific B‐cell subsets and enhancement of anti‐tumor B‐cell subsets, or combination with ICI, would also be potent therapeutic options.^[^ ^155^ ^]^ A DART therapy (MGD010), which has recently been in development, can inhibit autoimmunity by disrupting BCR signaling by targeting FcγRIIb and Igβ on B cells.^[^ ^156^ ^]^ B‐cell ICI may provide new ideas for the development of bispecific antibodies in tumors. Thus, does B‐cell ICI in combination with other immunotherapies present better efficacy and improve clinical outcomes in tumors? If so, what is the rationale and therapeutic basis for this combination therapy? In the future, not only is it necessary to explore the therapeutic effects of B‐cell ICI in clinical tumor patients, but it is equally important to study the clinical efficacy of B‐cell ICI in combination with other immunotherapies, which may bring new breakthroughs in tumor therapy.

This article is the first to explore the role of B‐cell ICs in tumor immune progression. B‐cell ICs are anticipated to serve as a novel target for tumor immunotherapy. High expression of ICs in B cells contributes to B‐cell dysfunction and fosters an immunosuppressive TME. Targeting B‐cell ICs may help to restore B‐cell functions of proliferation, activation, antigen presentation and antibody secretion, and to release the limitation of effector T cells, as well as activate humoral immune responses. B‐cell ICs have been demonstrated to be a crucial component of the tumor immune response. These findings contribute to a deeper understanding of the relationship between B‐cell ICs and tumor immunity, broaden the application of ICI in tumors, and provide new hope for tumor immunotherapy.

## Conflict of Interest

The authors declare no conflict of interest.

## Author Contributions

X.Y.S., X.S.C., A.M.J., and W.J.S. contributed equally to this work and share first authorship. P.L., L.H.W., A.Q.L., and Q.C. conceived the idea, provided constructive guidance, and supervised the study. X.Y.S. wrote the manuscript and visualized the figures. X.Y.S., A.Q.L., X.S.C., A.M.J., and W.J.S. corrected the manuscript and figures. L.X.Z., W.M.M., A.G., and Z.Q.L. revised the manuscript. All authors reviewed and edited the manuscript, contributed to the article, and approved the submitted version.

## References

[1] J. R. Conejo‐Garcia , S. Biswas , R. Chaurio , P. C. Rodriguez , Semin. Immunol. 2023, 65, 101707. 36527759 10.1016/j.smim.2022.101707 PMC10123518

[2] B. Mirlekar , D. Michaud , S. J. Lee , N. P. Kren , C. Harris , K. Greene , E. C. Goldman , G. P. Gupta , R. C. Fields , W. G. Hawkins , D. G. DeNardo , N. U. Rashid , J. J. Yeh , A. J. McRee , B. G. Vincent , D. A. A. Vignali , Y. Pylayeva‐Gupta , Cancer Immunol. Res. 2020, 8, 292. 32024640 10.1158/2326-6066.CIR-19-0349 PMC7056532

[3] H. T. Hu , X. Ai , M. Lu , Z. Song , H. Li , Exp. Cell Res. 2019, 384, 111652. 31574287 10.1016/j.yexcr.2019.111652

[4] G. Huai , J. F. Markmann , S. Deng , C. G. Rickert , Clin. Transl. Immunol. 2021, 10, 1270. 10.1002/cti2.1270 PMC8017464 33815797

[5] C. M. Laumont , A. C. Banville , M. Gilardi , D. P. Hollern , B. H. Nelson , Nat. Rev. Cancer 2022, 22, 414. 35393541 10.1038/s41568-022-00466-1 PMC9678336

[6] M. Mizukami , T. Hanagiri , M. Yasuda , K. Kuroda , Y. Shigematsu , T. Baba , T. Fukuyama , Y. Nagata , T. So , Y. Ichiki , M. Sugaya , T. So , M. Takenoyama , K. Sugio , K. Yasumoto , Cancer Res. 2007, 67, 8351. 17804751 10.1158/0008-5472.CAN-06-3889

[7] K. C. Foy , R. M. Wygle , M. J. Miller , J. P. Overholser , T. Bekaii‐Saab , P. T. Kaumaya , J. Immunol. 2013, 191, 217. 23698748 10.4049/jimmunol.1300231 PMC4324564

[8] M. Candolfi , J. F. Curtin , K. Yagiz , H. Assi , M. K. Wibowo , G. E. Alzadeh , D. Foulad , A. G. Muhammad , S. Salehi , N. Keech , M. Puntel , C. Liu , N. R. Sanderson , K. M. Kroeger , R. Dunn , G. Martins , P. R. Lowenstein , M. G. Castro , Neoplasia 2011, 13, 947. 22028620 10.1593/neo.11024 PMC3201571

[9] V. Engelhard , J. R. Conejo‐Garcia , R. Ahmed , B. H. Nelson , K. Willard‐Gallo , T. C. Bruno , W. H. Fridman , Cancer Cell 2021, 39, 1293. 34597591 10.1016/j.ccell.2021.09.007

[10] K. Hladíková , V. Koucký , J. Bouček , J. Laco , M. Grega , M. Hodek , M. Zábrodský , M. Vošmik , K. Rozkošová , H. Vošmiková , P. Čelakovský , V. Chrobok , A. Ryška , R. Špíšek , A. Fialová , J. Immunother. Cancer 2019, 7, 261. 31623665 10.1186/s40425-019-0726-6 PMC6796441

[11] X. Hu , X. S. Liu , Immunity 2022, 55, 387. 35263565 10.1016/j.immuni.2022.02.009

[12] W. H. Fridman , M. Meylan , F. Petitprez , C. M. Sun , A. Italiano , Nat. Rev. Clin. Oncol. 2022, 19, 441. 35365796 10.1038/s41571-022-00619-z

[13] M. Meylan , F. Petitprez , E. Becht , A. Bougoüin , G. Pupier , A. Calvez , I. Giglioli , V. Verkarre , G. Lacroix , J. Verneau , C.‐M. Sun , P. Laurent‐Puig , Y.‐A. Vano , R. Elaïdi , A. Méjean , R. Sanchez‐Salas , E. Barret , X. Cathelineau , S. Oudard , C.‐A. Reynaud , A. de Reyniès , C. Sautès‐Fridman , W. H. Fridman , Immunity. 2022, 55, 527. 35231421 10.1016/j.immuni.2022.02.001

[14] G. S. Kinker , G. A. F. Vitiello , A. B. Diniz , M. P. Cabral‐Piccin , P. H. B. Pereira , M. L. R. Carvalho , W. A. S. Ferreira , A. S. Chaves , A. Rondinelli , A. F. Gusmão , A. Defelicibus , G. O. dos Santos , W. A. Nunes , L. C. L. Claro , T. M. Bernardo , R. T. Nishio , A. M. Pacheco , A. C. Laus , L. M. R. B. Arantes , J. L. Fleck , V. H. F. de Jesus , A. de Moricz , R. Weinlich , F. J. F. Coimbra , V. C. C. de Lima , T. D. S. Medina , Gut 2023, 72, 1927. 37230755 10.1136/gutjnl-2022-328697

[15] K. T. Lynch , S. J. Young , M. O. Meneveau , N. A. Wages , V. H. Engelhard , C. L. Slingluff Jr. , I. S. Mauldin , J. Immunother. Cancer 2021, 9, 002273. 10.1136/jitc-2020-002273 PMC8190052 34103353

[16] C. Robert , Nat. Commun. 2020, 11, 3801. 32732879 10.1038/s41467-020-17670-y PMC7393098

[17] S. Bagchi , R. Yuan , E. G. Engleman , Annu. Rev. Pathol. 2021, 16, 223. 33197221 10.1146/annurev-pathol-042020-042741

[18] X. He , C. Xu , Cell Res. 2020, 30, 660. 32467592 10.1038/s41422-020-0343-4 PMC7395714

[19] E. A. Tivol , F. Borriello , A. N. Schweitzer , W. P. Lynch , J. A. Bluestone , A. H. Sharpe , Immunity 1995, 3, 541. 7584144 10.1016/1074-7613(95)90125-6

[20] M. Khan , S. Arooj , H. Wang , Front. Immunol. 2020, 11, 167. 32117298 10.3389/fimmu.2020.00167 PMC7031489

[21] S. Xu , C. Wang , L. Yang , J. Wu , M. Li , P. Xiao , Z. Xu , Y. Xu , K. Wang , Front. Immunol. 2023, 14, 1199631. 37313405 10.3389/fimmu.2023.1199631 PMC10258331

[22] Q. Zhang , J. Bi , X. Zheng , Y. Chen , H. Wang , W. Wu , Z. Wang , Q. Wu , H. Peng , H. Wei , R. Sun , Z. Tian , Nat. Immunol. 2018, 19, 723. 29915296 10.1038/s41590-018-0132-0

[23] Y. Mei , X. Wang , J. Zhang , D. Liu , J. He , C. Huang , J. Liao , Y. Wang , Y. Feng , H. Li , X. Liu , L. Chen , W. Yi , X. Chen , H.‐M. Bai , X. Wang , Y. Li , L. Wang , Z. Liang , X. Ren , L. Qiu , Y. Hui , Q. Zhang , Q. Leng , J. Chen , G. Jia , Nat. Cancer. 2023, 4, 1273. 37460871 10.1038/s43018-023-00598-9

[24] S. Serrati , F. Margheri , Biomolecules 2023, 13, 1209. 37627274 10.3390/biom13081209 PMC10452670

[25] J. Liu , Z. Chen , Y. Li , W. Zhao , J. Wu , Z. Zhang , Front. Pharmacol. 2021, 12, 731798. 34539412 10.3389/fphar.2021.731798 PMC8440961

[26] L. Bod , Y.‐C. Kye , J. Shi , E. Torlai Triglia , A. Schnell , J. Fessler , S. M. Ostrowski , M. Y. Von‐Franque , J. R. Kuchroo , R. M. Barilla , S. Zaghouani , E. Christian , T. M. Delorey , K. Mohib , S. Xiao , N. Slingerland , C. J. Giuliano , O. Ashenberg , Z. Li , D. M. Rothstein , D. E. Fisher , O. Rozenblatt‐Rosen , A. H. Sharpe , F. J. Quintana , L. Apetoh , A. Regev , V. K. Kuchroo , Nature 2023, 619, 348. 37344597 10.1038/s41586-023-06231-0 PMC10795478

[27] A. Herrmann , C. Lahtz , T. Nagao , J. Y. Song , W. C. Chan , H. Lee , C. Yue , T. Look , R. Mülfarth , W. Li , K. Jenkins , J. Williams , L. E. Budde , S. Forman , L. Kwak , T. Blankenstein , H. Yu , Cancer Res. 2017, 77, 5118. 28716895 10.1158/0008-5472.CAN-16-0342 PMC5600851

[28] H. Liu , J. Wu , X. Xu , H. Wang , C. Zhang , S. Yin , Y. He , Int. Immunopharmacol. 2022, 108, 108735. 35405596 10.1016/j.intimp.2022.108735

[29] P. B. Olkhanud , B. Damdinsuren , M. Bodogai , R. E. Gress , R. Sen , K. Wejksza , E. Malchinkhuu , R. P. Wersto , A. Biragyn , Cancer Res. 2011, 71, 3505. 21444674 10.1158/0008-5472.CAN-10-4316 PMC3096701

[30] M. T. A. Khan , C. Cervantes , S. Viswanadhapalli , Y. Hui , R. Vadlamudi , Z. Xu , Cancer Res. 2022, 82, 10.1158/1538-7445.SABCS21-P1-04-10.

[31] X. Tian , X. Zheng , D. Tian , Signal Transduction Targeted Ther. 2023, 8, 389. 10.1038/s41392-023-01643-w PMC10587137 37857611

[32] J. Griss , W. Bauer , C. Wagner , M. Simon , M. Chen , K. Grabmeier‐Pfistershammer , M. Maurer‐Granofszky , F. Roka , T. Penz , C. Bock , G. Zhang , M. Herlyn , K. Glatz , H. Läubli , K. D. Mertz , P. Petzelbauer , T. Wiesner , M. Hartl , W. F. Pickl , R. Somasundaram , P. Steinberger , S. N. Wagner , Nat. Commun. 2019, 10, 4186. 31519915 10.1038/s41467-019-12160-2 PMC6744450

[33] K. Wejksza , C. Lee‐Chang , M. Bodogai , J. Bonzo , F. J. Gonzalez , E. Lehrmann , K. Becker , A. Biragyn , J. Immunol. 2013, 190, 2575. 23408836 10.4049/jimmunol.1201920 PMC3594535

[34] V. Rebmann , L. Konig , S. Nardi Fda , B. Wagner , L. F. Manvailer , P. A. Horn , Front. Immunol. 2016, 7, 173. 27199995 10.3389/fimmu.2016.00173 PMC4854879

[35] A. Lin , W. H. Yan , Mol. Med. 2015, 21, 782. 26322846 10.2119/molmed.2015.00083 PMC4749493

[36] A. Naji , C. Menier , F. Morandi , S. Agaugué , G. Maki , E. Ferretti , S. Bruel , V. Pistoia , E. D. Carosella , N. Rouas‐Freiss , J. Immunol. 2014, 192, 1536. 24453251 10.4049/jimmunol.1300438

[37] P. Vaupel , M. Hockel , A. Mayer , Antioxid. Redox Signaling 2007, 9, 1221. 10.1089/ars.2007.1628 17536958

[38] J. Zhang , X. Wu , J. Ma , K. Long , J. Sun , M. Li , L. Ge , Front. Immunol. 2022, 13, 967576. 36045669 10.3389/fimmu.2022.967576 PMC9421003

[39] Y. Li , L. Zhao , X. F. Li , Technol. Cancer Res. Treat. 2021, 20, 15330338211036304. 34350796 10.1177/15330338211036304 PMC8358492

[40] S. Jiang , R. Feng , Z. Tian , J. Zhou , W. Zhang , Cancer Lett. 2023, 556, 216076. 36724837 10.1016/j.canlet.2023.216076

[41] Y. Wang , C. C. Schafer , K. P. Hough , S. Tousif , S. R. Duncan , J. F. Kearney , S. Ponnazhagan , H.‐C. Hsu , J. S. Deshane , J. Immunol. 2018, 201, 278. 29752311 10.4049/jimmunol.1701069 PMC6008229

[42] F. J. N. Lelis , J. Jaufmann , A. Singh , K. Fromm , A. C. Teschner , S. Pöschel , I. Schäfer , S. Beer‐Hammer , N. Rieber , D. Hartl , Immunol. Lett. 2017, 188, 108. 28687234 10.1016/j.imlet.2017.07.003

[43] D. E. Kennedy , K. L. Knight , J. Immunol. 2015, 195, 2666. 26268654 10.4049/jimmunol.1500957 PMC4561202

[44] B. B. Cazac , J. Roes , Immunity 2000, 13, 443. 11070163 10.1016/s1074-7613(00)00044-3

[45] M. K.‐K. Chan , J. Y.‐F. Chung , P. C.‐T. Tang , A. S.‐W. Chan , J. Y.‐Y. Ho , T. P.‐T. Lin , J. Chen , K.‐T. Leung , K.‐F. To , H.‐Y. Lan , P. M.‐K. Tang , Cancer Lett. 2022, 550, 215925. 36183857 10.1016/j.canlet.2022.215925

[46] M. Bodogai , C. L. Chang , K. Wejksza , J. Lai , M. Merino , R. P. Wersto , R. E. Gress , A. C. Chan , C. Hesdorffer , A. Biragyn , Cancer Res. 2013, 73, 2127. 23365136 10.1158/0008-5472.CAN-12-4184 PMC3618504

[47] E. Ragonnaud , K. Moritoh , M. Bodogai , F. Gusev , S. Garaud , C. Chen , X. Wang , T. Baljinnyam , K. G. Becker , R. W. Maul , K. Willard‐Gallo , E. Rogaev , A. Biragyn , Cancer Res. 2019, 79, 5826. 31575547 10.1158/0008-5472.CAN-19-1058 PMC6881554

[48] C. Chen , B. Park , E. Ragonnaud , M. Bodogai , X. Wang , L. Zong , J.‐M. Lee , I. Beerman , A. Biragyn , Nat. Commun. 2022, 13, 5376. 36104343 10.1038/s41467-022-33117-y PMC9474882

[49] L. Ye , Q. Zhang , Y. Cheng , X. Chen , G. Wang , M. Shi , T. Zhang , Y. Cao , H. Pan , L. Zhang , G. Wang , Y. Deng , Y. Yang , G. Chen , J. Immunother. Cancer 2018, 6, 145. 30526680 10.1186/s40425-018-0451-6 PMC6288912

[50] X. Xiao , X.‐M. Lao , M.‐M. Chen , R.‐X. Liu , Y. Wei , F.‐Z. Ouyang , D.‐P. Chen , X.‐Y. Zhao , Q. Zhao , X.‐F. Li , C.‐L. Liu , L. Zheng , D.‐M. Kuang , Cancer Discovery 2016, 6, 546. 26928313 10.1158/2159-8290.CD-15-1408

[51] M. Shen , J. Wang , W. Yu , C. Zhang , M. Liu , K. Wang , L. Yang , F. Wei , S. E. Wang , Q. Sun , X. Ren , Oncoimmunology 2018, 7, 1413520. 10.1080/2162402X.2017.1413520 PMC5889195 29632731

[52] M. Liu , F. Wei , J. Wang , W. Yu , M. Shen , T. Liu , D. Zhang , Y. Wang , X. Ren , Q. Sun , Cell Death Dis. 2021, 12, 465. 33967272 10.1038/s41419-021-03745-1 PMC8107179

[53] C. Zhang , H. Xin , W. Zhang , P. J. Yazaki , Z. Zhang , K. Le , W. Li , H. Lee , L. Kwak , S. Forman , R. Jove , H. Yu , Immunity 2016, 44, 913. 27096320 10.1016/j.immuni.2016.04.003 PMC4844010

[54] Z. Lu , R. Liu , Y. Wang , M. Jiao , Z. Li , Z. Wang , C. Huang , G. Shi , A. Ke , L. Wang , Y. Fu , J. Xia , H. Wen , J. Zhou , X. Wang , D. Ye , J. Fan , Y. Chu , J. Cai , Hepatology 2023, 77, 745. 35243663 10.1002/hep.32442

[55] C. Cai , J. Zhang , M. Li , Z.‐J. Wu , K. H. Song , T. W. Zhan , L.‐H. Wang , Y.‐H. Sun , Tumour Biol. 2016, 37, 8209. 26715281 10.1007/s13277-015-4687-1

[56] M. Zhou , Z. Wen , F. Cheng , J. Ma , W. Li , H. Ren , Y. Sheng , H. Dong , L. Lu , H.‐M. Hu , L.‐X. Wang , Oncoimmunology 2016, 5, 1180485. 10.1080/2162402X.2016.1180485 PMC5006924 27622036

[57] H. He , J. Wu , M. Zang , W. Wang , X. Chang , X. Chen , R. Wang , Z. Wu , L. Wang , D. Wang , F. Lu , Z. Sun , C. Qu , Am. J. Cancer Res. 2017, 7, 1151. 28560063 PMC5446480

[58] B. Zhang , A. Vogelzang , M. Miyajima , Y. Sugiura , Y. Wu , K. Chamoto , R. Nakano , R. Hatae , R. J. Menzies , K. Sonomura , N. Hojo , T. Ogawa , W. Kobayashi , Y. Tsutsui , S. Yamamoto , M. Maruya , S. Narushima , K. Suzuki , H. Sugiya , K. Murakami , M. Hashimoto , H. Ueno , T. Kobayashi , K. Ito , T. Hirano , K. Shiroguchi , F. Matsuda , M. Suematsu , T. Honjo , S. Fagarasan , Nature 2021, 599, 471. 34732892 10.1038/s41586-021-04082-1 PMC8599023

[59] K. de Jonge , L. Tillé , J. Lourenco , H. Maby‐El Hajjami , S. Nassiri , J. Racle , D. Gfeller , M. Delorenzi , G. Verdeil , P. Baumgaertner , D. E. Speiser , Oncoimmunology 2021, 10, 1873585. 33643691 10.1080/2162402X.2021.1873585 PMC7872097

[60] M. Bodogai , K. Moritoh , C. Lee‐Chang , C. M. Hollander , C. A. Sherman‐Baust , R. P. Wersto , Y. Araki , I. Miyoshi , L. Yang , G. Trinchieri , A. Biragyn , Cancer Res. 2015, 75, 3456. 26183924 10.1158/0008-5472.CAN-14-3077 PMC4558269

[61] J. Eckl‐Dorna , F. D. Batista , Blood 2009, 113, 3969. 19144984 10.1182/blood-2008-10-185421

[62] P. Tolar , J. Hanna , P. D. Krueger , S. K. Pierce , Immunity 2009, 30, 44. 19135393 10.1016/j.immuni.2008.11.007 PMC2656684

[63] G. V. Sharonov , E. O. Serebrovskaya , D. V. Yuzhakova , O. V. Britanova , D. M. Chudakov , Nat. Rev. Immunol. 2020, 20, 294. 31988391 10.1038/s41577-019-0257-x

[64] S. S. Jeske , M. Brand , A. Ziebart , S. Laban , J. Doescher , J. Greve , E. K. Jackson , T. K. Hoffmann , C. Brunner , P. J. Schuler , Cancer Immunol. Immunother. 2020, 69, 1205. 32146518 10.1007/s00262-020-02535-6 PMC7303082

[65] B. Mirlekar , Y. Wang , S. Li , M. Zhou , S. Entwistle , T. De Buysscher , A. Morrison , G. Herrera , C. Harris , B. G. Vincent , J. P.‐Y. Ting , N. Rashid , W. Y. Kim , J. J. Yeh , Y. Pylayeva‐Gupta , Cell Rep. Med. 2022, 3, 100744. 36099917 10.1016/j.xcrm.2022.100744 PMC9512696

[66] J. F. Cohen‐Solal , L. Cassard , E. M. Fournier , S. M. Loncar , W. H. Fridman , C. Sautes‐Fridman , Dermatol. Res. Pract. 2010, 2010, 657406. 20672001 10.1155/2010/657406 PMC2905727

[67] R. A. Clynes , T. L. Towers , L. G. Presta , J. V. Ravetch , Nat. Med. 2000, 6, 443. 10742152 10.1038/74704

[68] F. Galvez‐Cancino , A. P. Simpson , C. Costoya , I. Matos , D. Qian , K. S. Peggs , K. Litchfield , S. A. Quezada , Nat. Rev. Cancer 2024, 24, 51. 38062252 10.1038/s41568-023-00637-8

[69] P. Andreu , M. Johansson , N. I. Affara , F. Pucci , T. Tan , S. Junankar , L. Korets , J. Lam , D. Tawfik , D. G. DeNardo , L. Naldini , K. E. de Visser , M. De Palma , L. M. Coussens , Cancer Cell 2010, 17, 121. 20138013 10.1016/j.ccr.2009.12.019 PMC3082507

[70] M. A. Spurrier , J. E. Jennings‐Gee , C. A. Daly , K. M. Haas , J. Immunol. 2021, 207, 1978. 34535576 10.4049/jimmunol.2100336 PMC8492549

[71] Y. Zhang , P. Chen , Cell. Mol. Biol. 2022, 68, 43. 10.14715/cmb/2022.68.11.8 37114308

[72] P. P. Domeier , S. B. Chodisetti , S. L. Schell , Y. I. Kawasawa , M. J. Fasnacht , C. Soni , Z. S. M. Rahman , Cell Rep. 2018, 24, 406. 29996101 10.1016/j.celrep.2018.06.046 PMC6089613

[73] I. Rastogi , D. Jeon , J. E. Moseman , A. Muralidhar , H. K. Potluri , D. G. McNeel , Front. Immunol. 2022, 13, 954936. 36159874 10.3389/fimmu.2022.954936 PMC9493130

[74] C. Ghosh , G. Luong , Y. Sun , J. Cancer 2021, 12, 2735. 33854633 10.7150/jca.57334 PMC8040720

[75] X. Wang , G. Wang , Z. Wang , B. Liu , N. Han , J. Li , C. Lu , X. Liu , Q. Zhang , Q. Yang , G. Wang , Mol. Immunol. 2019, 109, 20. 30851633 10.1016/j.molimm.2019.02.009

[76] M.‐L. Thibult , E. Mamessier , J. Gertner‐Dardenne , S. Pastor , S. Just‐Landi , L. Xerri , B. Chetaille , D. Olive , Int. Immunol. 2013, 25, 129. 23087177 10.1093/intimm/dxs098

[77] T. Okazaki , A. Maeda , H. Nishimura , T. Kurosaki , T. Honjo , Proc. Natl. Acad. Sci. USA 2001, 98, 13866. 11698646 10.1073/pnas.231486598 PMC61133

[78] B. A. Helmink , S. M. Reddy , J. Gao , S. Zhang , R. Basar , R. Thakur , K. Yizhak , M. Sade‐Feldman , J. Blando , G. Han , V. Gopalakrishnan , Y. Xi , H. Zhao , R. N. Amaria , H. A. Tawbi , A. P. Cogdill , W. Liu , V. S. LeBleu , F. G. Kugeratski , S. Patel , M. A. Davies , P. Hwu , J. E. Lee , J. E. Gershenwald , A. Lucci , R. Arora , S. Woodman , E. Z. Keung , P.‐O. Gaudreau , A. Reuben , et al., Nature 2020, 577, 549. 31942075 10.1038/s41586-019-1922-8 PMC8762581

[79] Y. Zhang , R. Morgan , C. Chen , Y. Cai , E. Clark , W. N. Khan , S.‐U. Shin , H.‐M. Cho , A. Al Bayati , A. Pimentel , J. D. Rosenblatt , Int. Immunol. 2016, 28, 423. 26895637 10.1093/intimm/dxw007 PMC5006091

[80] D.‐N. Tong , J. Guan , J.‐H. Sun , C.‐Y. Zhao , S.‐G. Chen , Z.‐Y. Zhang , Z.‐Q. Zhou , Clin. Exp. Pharmacol. Physiol. 2020, 47, 1342.32248559

[81] S. Van Coillie , B. Wiernicki , J. Xu , Adv. Exp. Med. Biol. 2020, 1248, 7. 32185705 10.1007/978-981-15-3266-5_2

[82] J. M. Chauvin , H. M. Zarour , J. Immunother. Cancer 2020, 8, 000957. 10.1136/jitc-2020-000957 PMC7477968 32900861

[83] R. J. Harris , Z. Willsmore , R. Laddach , S. Crescioli , J. Chauhan , A. Cheung , A. Black , J. L. C. Geh , A. D. MacKenzie Ross , C. Healy , S. Tsoka , J. Spicer , K. E. Lacy , S. N. Karagiannis , Tregs. Oncoimmunol. 2022, 11, 2104426. 10.1080/2162402X.2022.2104426 PMC9336482 35909944

[84] Y. Zhang , R. Morgan , E. R. Podack , J. Rosenblatt , Immunol. Res. 2013, 57, 115. 24293009 10.1007/s12026-013-8472-1

[85] S. Inoue , W. W. Leitner , B. Golding , D. Scott , Cancer Res. 2006, 66, 7741. 16885377 10.1158/0008-5472.CAN-05-3766

[86] E. Boggio , C. L. Gigliotti , R. Moia , A. Scotta , I. Crespi , P. Boggione , L. De Paoli , C. Deambrogi , M. Garzaro , M. Vidali , A. Chiocchetti , I. Stoppa , R. Rolla , C. Dianzani , C. Monge , N. Clemente , G. Gaidano , U. Dianzani , Br. J. Haematol. 2022, 196, 1369. 34954822 10.1111/bjh.17968

[87] X. Lu , Curr. Med. Chem. 2021, 28, 5659. 33372866 10.2174/0929867328666201229123151

[88] B. O. Lee , J. Moyron‐Quiroz , J. Rangel‐Moreno , K. L. Kusser , L. Hartson , F. Sprague , F. E. Lund , T. D. Randall , J. Immunol. 2003, 171, 5707. 14634078 10.4049/jimmunol.171.11.5707

[89] W. Luo , L. Conter , R. A. Elsner , S. Smita , F. Weisel , D. Callahan , S. Wu , M. Chikina , M. Shlomchik , Sci. Immunol. 2023, 8, add1823. 10.1126/sciimmunol.add1823 PMC10206726 36800413

[90] J. Ren , T. Lan , T. Liu , Y. Liu , B. Shao , K. Men , Y. Ma , X. Liang , Y.‐Q. Wei , M. Luo , X.‐W. Wei , J. Immunol. 2022, 208, 2425. 35437281 10.4049/jimmunol.2100341 PMC9125199

[91] C.‐H. Hsieh , C.‐Z. Jian , L.‐I. Lin , G.‐S. Low , P.‐Y. Ou , C. Hsu , D.‐L. Ou , Cancers (Basel) 2022, 14, 294. 35053457 10.3390/cancers14020294 PMC8774093

[92] S. Chikuma , Curr. Top. Microbiol. Immunol. 2017, 410, 99. 28900679 10.1007/82_2017_61

[93] K. E. Pauken , J. A. Torchia , A. Chaudhri , A. H. Sharpe , G. J. Freeman , Semin. Immunol. 2021, 52, 101480. 34006473 10.1016/j.smim.2021.101480 PMC8545711

[94] M. Heiduk , A. Klimova , C. Reiche , D. Digomann , C. Beer , D. E. Aust , M. Distler , J. Weitz , A. M. Seifert , L. Seifert , Clin. Cancer Res. 2023, 29, 2638. 37140899 10.1158/1078-0432.CCR-23-0258 PMC10345964

[95] M. Yi , M. Niu , L. Xu , S. Luo , K. Wu , J. Hematol. Oncol. 2021, 14, 10. 33413496 10.1186/s13045-020-01027-5 PMC7792099

[96] Y. Iwai , M. Ishida , Y. Tanaka , T. Okazaki , T. Honjo , N. Minato , Proc. Natl. Acad. Sci. USA 2002, 99, 12293. 12218188 10.1073/pnas.192461099 PMC129438

[97] D. Ostroumov , S. Duong , J. Wingerath , N. Woller , M. P. Manns , K. Timrott , M. Kleine , W. Ramackers , S. Roessler , S. Nahnsen , S. Czemmel , O. Dittrich‐Breiholz , T. Eggert , F. Kühnel , T. C. Wirth , Hepatology 2021, 73, 1399. 32716559 10.1002/hep.31466

[98] T. Yokosuka , M. Takamatsu , W. Kobayashi‐Imanishi , A. Hashimoto‐Tane , M. Azuma , T. Saito , J. Exp. Med. 2012, 209, 1201. 22641383 10.1084/jem.20112741 PMC3371732

[99] X. Xu , B. Hou , A. Fulzele , T. Masubuchi , Y. Zhao , Z. Wu , Y. Hu , Y. Jiang , Y. Ma , H. Wang , E. J. Bennett , G. Fu , E. Hui , J. Cell Biol. 2020, 219, 201905085. 10.1083/jcb.201905085 PMC7265324 32437509

[100] F. Chen , Y. Xu , Y. Chen , S. Shan , Cancer Med. 2020, 9, 3584. 32212317 10.1002/cam4.2976 PMC7221438

[101] R. J. Johnston , L. Comps‐Agrar , J. Hackney , X. Yu , M. Huseni , Y. Yang , S. Park , V. Javinal , H. Chiu , B. Irving , D. L. Eaton , J. L. Grogan , Cancer Cell 2014, 26, 923. 25465800 10.1016/j.ccell.2014.10.018

[102] K. L. Banta , X. Xu , A. S. Chitre , A. Au‐Yeung , C. Takahashi , W. E. O'Gorman , T. D. Wu , S. Mittman , R. Cubas , L. Comps‐Agrar , A. Fulzele , E. J. Bennett , J. L. Grogan , E. Hui , E. Y. Chiang , I. Mellman , Immunity 2022, 55, 512. 35263569 10.1016/j.immuni.2022.02.005 PMC9287124

[103] S. Ding , N. Qiao , Q. Zhu , Y. Tong , S. Wang , X. Chen , Q. Tian , Y. Xiao , K. Shen , Cancer Commun. (Lond) 2023, 43, 661. 37158690 10.1002/cac2.12429 PMC10259667

[104] K. Wennhold , M. Thelen , J. Lehmann , S. Schran , E. Preugszat , M. Garcia‐Marquez , A. Lechner , A. Shimabukuro‐Vornhagen , M. S. Ercanoglu , F. Klein , F. Thangarajah , S. Eidt , H. Löser , C. Bruns , A. Quaas , M. von Bergwelt‐Baildon , H. A. Schlößer , Cancer Immunol. Res. 2021, 9, 1098. 34155067 10.1158/2326-6066.CIR-20-0949

[105] A. Jo , D. Jeong , H. H. Eum , N. Kim , M. Na , H. Kang , H.‐O. Lee , Int. J. Cancer 2023, 152, 1964. 36650700 10.1002/ijc.34438

[106] J.‐C. Guo , C.‐L. Hsu , Y.‐L. Huang , C.‐C. Lin , T.‐C. Huang , I.‐C. Wu , C.‐Y. Lin , M.‐Y. Lien , H.‐Y. Kuo , A.‐L. Cheng , C.‐H. Hsu , Front. Oncol. 2022, 12, 879398. 35847892 10.3389/fonc.2022.879398 PMC9276977

[107] S. Biswas , G. Mandal , K. K. Payne , C. M. Anadon , C. D. Gatenbee , R. A. Chaurio , T. L. Costich , C. Moran , C. M. Harro , K. E. Rigolizzo , J. A. Mine , J. Trillo‐Tinoco , N. Sasamoto , K. L. Terry , D. Marchion , A. Buras , R. M. Wenham , X. Yu , M. K. Townsend , S. S. Tworoger , P. C. Rodriguez , A. R. Anderson , J. R. Conejo‐Garcia , Nature 2021, 591, 464. 33536615 10.1038/s41586-020-03144-0 PMC7969354

[108] S. S. Kim , S. Shen , S. Miyauchi , P. D. Sanders , I. Franiak‐Pietryga , L. Mell , J. S. Gutkind , E. E. W. Cohen , J. A. Califano , A. B. Sharabi , Clin. Cancer Res. 2020, 26, 3345. 32193227 10.1158/1078-0432.CCR-19-3211 PMC7334097

[109] N. S. Patil , B. Y. Nabet , S. Müller , H. Koeppen , W. Zou , J. Giltnane , A. Au‐Yeung , S. Srivats , J. H. Cheng , C. Takahashi , P. E. de Almeida , A. S. Chitre , J. L. Grogan , L. Rangell , S. Jayakar , M. Peterson , A. W. Hsia , W. E. O'Gorman , M. Ballinger , R. Banchereau , D. S. Shames , Cancer Cell 2022, 40, 289. 35216676 10.1016/j.ccell.2022.02.002

[110] L. A. Dempsey , Nat. Immunol. 2023, 24, 1211. 10.1038/s41590-023-01582-2 37488433

[111] F. Petitprez , A. de Reyniès , E. Z. Keung , T. W.‐W. Chen , C.‐M. Sun , J. Calderaro , Y.‐M. Jeng , L.‐P. Hsiao , L. Lacroix , A. Bougoüin , M. Moreira , G. Lacroix , I. Natario , J. Adam , C. Lucchesi , Y. H. Laizet , M. Toulmonde , M. A. Burgess , V. Bolejack , D. Reinke , K. M. Wani , W.‐L. Wang , A. J. Lazar , C. L. Roland , J. A. Wargo , A. Italiano , C. Sautès‐Fridman , H. A. Tawbi , W. H. Fridman , Nature 2020, 577, 556. 31942077 10.1038/s41586-019-1906-8

[112] Cancer Discovery 2022, 12, 1179.

[113] A. T. Ruffin , A. R. Cillo , T. Tabib , A. Liu , S. Onkar , S. R. Kunning , C. Lampenfeld , H. I. Atiya , I. Abecassis , C. H. L. Kürten , Z. Qi , R. Soose , U. Duvvuri , S. Kim , S. Oesterrich , R. Lafyatis , L. G. Coffman , R. L. Ferris , D. A. A. Vignali , T. C. Bruno , Nat. Commun. 2021, 12, 3349. 34099645 10.1038/s41467-021-23355-x PMC8184766

[114] W. H. Fridman , S. Siberil , G. Pupier , S. Soussan , C. Sautes‐Fridman , Semin. Immunol. 2023, 65, 101703. 36481358 10.1016/j.smim.2022.101703

[115] E. Playoust , R. Remark , E. Vivier , P. Milpied , Cell Mol. Immunol. 2023, 20, 1040. 37419983 10.1038/s41423-023-01060-7 PMC10468534

[116] A. B. Rodriguez , J. D. Peske , A. N. Woods , K. M. Leick , I. S. Mauldin , M. O. Meneveau , S. J. Young , R. S. Lindsay , M. M. Melssen , S. Cyranowski , G. Parriott , M. R. Conaway , Y.‐X. Fu , C. L. Slingluff , V. H. Engelhard , Cell Rep. 2021, 36, 109422. 34289373 10.1016/j.celrep.2021.109422 PMC8362934

[117] D. P. Hollern , N. Xu , A. Thennavan , C. Glodowski , S. Garcia‐Recio , K. R. Mott , X. He , J. P. Garay , K. Carey‐Ewend , D. Marron , J. Ford , S. Liu , S. C. Vick , M. Martin , J. S. Parker , B. G. Vincent , J. S. Serody , C. M. Perou , Cell 2019, 179, 1191. 31730857 10.1016/j.cell.2019.10.028 PMC6911685

[118] S. Sánchez‐Alonso , G. Setti‐Jerez , M. Arroyo , T. Hernández , M. I. Martos , J. M. Sánchez‐Torres , R. Colomer , A. R. Ramiro , A. Alfranca , J. Immunother. Cancer 2020, 8, 001187. 10.1136/jitc-2020-001187 PMC7478024 32900863

[119] I. Plesca , A. Tunger , L. Müller , R. Wehner , X. Lai , M.‐O. Grimm , S. Rutella , M. Bachmann , M. Schmitz , Front. Immunol. 2020, 11, 364. 32194568 10.3389/fimmu.2020.00364 PMC7064638

[120] N. Gavrielatou , E. Fortis , A. Spathis , M. Anastasiou , P. Economopoulou , G. R. P. Foukas , I. M. Lelegiannis , S. Rusakiewicz , I. Vathiotis , T. N. Aung , S. Tissot , A. Kastrinou , I. Kotsantis , E. M. Vagia , I. Panayiotides , D. L. Rimm , G. Coukos , K. Homicsko , P. Foukas , A. Psyrri , Ann. Oncol. 2024, 35, 340. 38159908 10.1016/j.annonc.2023.12.011

[121] R. Cabrita , M. Lauss , A. Sanna , M. Donia , M. Skaarup Larsen , S. Mitra , I. Johansson , B. Phung , K. Harbst , J. Vallon‐Christersson , A. van Schoiack , K. Lövgren , S. Warren , K. Jirström , H. Olsson , K. Pietras , C. Ingvar , K. Isaksson , D. Schadendorf , H. Schmidt , L. Bastholt , A. Carneiro , J. A. Wargo , I. M. Svane , G. Jönsson , Nature 2020, 577, 561. 31942071 10.1038/s41586-019-1914-8

[122] Y. Zhang , H. Chen , H. Mo , X. Hu , R. Gao , Y. Zhao , B. Liu , L. Niu , X. Sun , X. Yu , Y. Wang , Q. Chang , T. Gong , X. Guan , T. Hu , T. Qian , B. Xu , F. Ma , Z. Zhang , Z. Liu , Cancer Cell 2021, 39, 1578. 34653365 10.1016/j.ccell.2021.09.010

[123] K. Uehara , K. Tanoue , K. Yamaguchi , H. Ohmura , M. Ito , Y. Matsushita , K. Tsuchihashi , S. Tamura , H. Shimokawa , T. Isobe , Y. Shibata , H. Ariyama , R. Tanaka , H. Kusaba , H. Yamamoto , Y. Oda , K. Akashi , E. Baba , Cancer Immunol. Immunother. 2023, 72, 3543. 37550428 10.1007/s00262-023-03505-4 PMC10991473

[124] L. Xia , L. Guo , J. Kang , Y. Yang , Y. Yao , W. Xia , R. Sun , S. Zhang , W. Li , Y. Gao , H. Chen , Z. Li , J. Yang , S. Lu , Y. Wang , Front Immunol. 2021, 12, 759217. 34899709 10.3389/fimmu.2021.759217 PMC8652218

[125] A. Varghese , S. Reddy , E. Cortes , S. Liu , K. Attwood , K. Odunsi , E. Zsiros , Gynecol. Oncol. 2021, 162, S191.

[126] J. Budczies , M. Kirchner , K. Kluck , D. Kazdal , J. Glade , M. Allgäuer , M. Kriegsmann , C.‐P. Heußel , F. J. Herth , H. Winter , M. Meister , T. Muley , S. Fröhling , S. Peters , B. Seliger , P. Schirmacher , M. Thomas , P. Christopoulos , A. Stenzinger , Oncoimmunology 2021, 10, 1860586. 33520406 10.1080/2162402X.2020.1860586 PMC7808386

[127] F. S. Varn , Y. Wang , C. Cheng , Oncoimmunology 2019, 8, 1513440. 10.1080/2162402X.2018.1513440 PMC6287894 30546953

[128] A. Lundberg , B. Li , R. Li , Br. J. Cancer 2022, 126, 899. 34921229 10.1038/s41416-021-01674-6 PMC8927337

[129] P. Setordzi , X. Chang , Z. Liu , Y. Wu , D. Zuo , Eur. J. Pharmacol. 2021, 895, 173867. 33460617 10.1016/j.ejphar.2021.173867

[130] J.‐M. Sun , L. Shen , M. A. Shah , P. Enzinger , A. Adenis , T. Doi , T. Kojima , J.‐P. Metges , Z. Li , S.‐B. Kim , B. C. Cho , W. Mansoor , S.‐H. Li , P. Sunpaweravong , M. A. Maqueda , E. Goekkurt , H. Hara , L. Antunes , C. Fountzilas , A. Tsuji , V. C. Oliden , Q. Liu , S. Shah , P. Bhagia , K. Kato , Lancet 2021, 398, 759. 34454674 10.1016/S0140-6736(21)01234-4

[131] D. M. O'Malley , M. Neffa , B. J. Monk , T. Melkadze , M. Huang , A. Kryzhanivska , I. Bulat , T. M. Meniawy , A. Bagameri , E. W. Wang , B. Doger de Speville Uribe , R. Hegg , W. Ortuzar Feliu , M. Ancukiewicz , I. Lugowska , J. Clin. Oncol. 2022, 40, 762. 34932394 10.1200/JCO.21.02067 PMC8887945

[132] E. D. Routh , M. G. Woodcock , W. Beckabir , S. P. Vensko , J. S. Serody , B. G. Vincent , J. Immunother. Cancer 2023, 11, 005848. 10.1136/jitc-2022-005848 PMC10008414 36882226

[133] A. Lechner , H. A. Schlößer , M. Thelen , K. Wennhold , S. I. Rothschild , R. Gilles , A. Quaas , O. G. Siefer , C. U. Huebbers , E. Cukuroglu , J. Göke , A. Hillmer , B. Gathof , M. F. Meyer , J. P. Klussmann , A. Shimabukuro‐Vornhagen , S. Theurich , D. Beutner , M. von Bergwelt‐Baildon , Oncoimmunology 2019, 8, 1535293. 30723574 10.1080/2162402X.2018.1535293 PMC6350680

[134] J. Nagasaki , Y. Togashi , Int. Immunol. 2022, 34, 563. 35460561 10.1093/intimm/dxac013

[135] Q. Wang , Y. Qin , B. Li , Cancer Lett. 2023, 559, 216043. 36584935 10.1016/j.canlet.2022.216043

[136] Y. Huang , A. Jia , Y. Wang , G. Liu , Immunology 2023, 168, 30. 36190809 10.1111/imm.13588

[137] T. C. Bruno , P. J. Ebner , B. L. Moore , O. G. Squalls , K. A. Waugh , E. B. Eruslanov , S. Singhal , J. D. Mitchell , W. A. Franklin , D. T. Merrick , M. D. McCarter , B. E. Palmer , J. A. Kern , J. E. Slansky , Cancer Immunol. Res. 2017, 5, 898. 28848053 10.1158/2326-6066.CIR-17-0075 PMC5788174

[138] Q. Hu , Y. Hong , P. Qi , G. Lu , X. Mai , S. Xu , X. He , Y. Guo , L. Gao , Z. Jing , J. Wang , T. Cai , Y. Zhang , Nat. Commun. 2021, 12, 2186. 33846305 10.1038/s41467-021-22300-2 PMC8042001

[139] J. Z. Wang , Y. H. Zhang , X. H. Guo , H. Y. Zhang , Y. Zhang , Int. Immunopharmacol. 2016, 36, 73. 27111515 10.1016/j.intimp.2016.04.018

[140] S. Gu , L. Qian , Y. Zhang , K. Chen , Y. Li , J. Wang , P. Wang , Biochim. Biophys. Acta Rev. Cancer 2021, 1876, 188632. 34626740 10.1016/j.bbcan.2021.188632

[141] R. A. O'Connor , B. R. Martinez , L. Koppensteiner , L. Mathieson , A. R. Akram , Front. Immunol. 2023, 14, 1221532. 37520560 10.3389/fimmu.2023.1221532 PMC10373066

[142] M. Fässler , S. Diem , J. Mangana , O. Hasan Ali , F. Berner , D. Bomze , S. Ring , R. Niederer , C. del Carmen Gil Cruz , C. I. Pérez Shibayama , M. Krolik , M. Siano , M. Joerger , M. Recher , L. Risch , S. Güsewell , M. Risch , D. E. Speiser , B. Ludewig , M. P. Levesque , R. Dummer , L. Flatz , J. Immunother. Cancer 2019, 7, 50. 30786924 10.1186/s40425-019-0523-2 PMC6383238

[143] S. Diem , M. Fässler , D. Bomze , O. H. Ali , F. Berner , R. Niederer , D. Hillmann , J. Mangana , M. P. Levesque , R. Dummer , L. Risch , M. Recher , M. Risch , L. Flatz , J. Immunother. 2019, 42, 89. 30768543 10.1097/CJI.0000000000000255

[144] J. DeFalco , M. Harbell , A. Manning‐Bog , G. Baia , A. Scholz , B. Millare , M. Sumi , D. Zhang , F. Chu , C. Dowd , P. Zuno‐Mitchell , D. Kim , Y. Leung , S. Jiang , X. Tang , K. S. Williamson , X. Chen , S. M. Carroll , G. Espiritu Santo , N. Haaser , N. Nguyen , E. Giladi , D. Minor , Y. C. Tan , J. B. Sokolove , L. Steinman , T. A. Serafini , G. Cavet , N. M. Greenberg , J. Glanville , et al., Clin. Immunol. 2018, 187, 37. 29031828 10.1016/j.clim.2017.10.002

[145] A. Goenka , F. Khan , B. Verma , P. Sinha , C. C. Dmello , M. P. Jogalekar , P. Gangadaran , B.‐C. Ahn , Cancer Commun. (Lond) 2023, 43, 525. 37005490 10.1002/cac2.12416 PMC10174093

[146] F. Tang , C. Barbacioru , Y. Wang , E. Nordman , C. Lee , N. Xu , X. Wang , J. Bodeau , B. B. Tuch , A. Siddiqui , K. Lao , M. A. Surani , Nat. Methods 2009, 6, 377. 19349980 10.1038/nmeth.1315

[147] W. C. Dougall , S. Kurtulus , M. J. Smyth , A. C. Anderson , Immunol. Rev. 2017, 276, 112. 28258695 10.1111/imr.12518

[148] S. R. Ali , J. J. Fong , A. F. Carlin , T. D. Busch , R. Linden , T. Angata , T. Areschoug , M. Parast , N. Varki , J. Murray , V. Nizet , A. Varki , J. Exp. Med. 2014, 211, 1231. 24799499 10.1084/jem.20131853 PMC4042635

[149] E. A. Ansa‐Addo , H.‐C. Huang , B. Riesenberg , S. Iamsawat , D. Borucki , M. H. Nelson , J. H. Nam , D. Chung , C. M. Paulos , B. Liu , X.‐Z. Yu , C. Philpott , P. H. Howe , Z. Li , Sci. Adv. 2020, 6, aaz3865. 10.1126/sciadv.aaz3865 PMC7259945 32523987

[150] A. Vuchkovska , D. G. Glanville , G. M. Scurti , M. I. Nishimura , P. White , A. T. Ulijasz , M. Iwashima , Immunology 2022, 166, 238. 35290663 10.1111/imm.13470 PMC11590682

[151] F. Wiede , K.‐H. Lu , X. Du , M. N. Zeissig , R. Xu , P. K. Goh , C. E. Xirouchaki , S. J. Hogarth , S. Greatorex , K. Sek , R. J. Daly , P. A. Beavis , P. K. Darcy , N. K. Tonks , T. Tiganis , Cancer Discovery 2022, 12, 752. 34794959 10.1158/2159-8290.CD-21-0694 PMC8904293

[152] D. Mittal , A. Lepletier , J. Madore , A. R. Aguilera , K. Stannard , S. J. Blake , V. L. J. Whitehall , C. Liu , M. L. Bettington , K. Takeda , G. V. Long , R. A. Scolyer , R. Lan , N. Siemers , A. Korman , M. W. L. Teng , R. J. Johnston , W. C. Dougall , M. J. Smyth , Cancer Immunol. Res. 2019, 7, 559. 30894377 10.1158/2326-6066.CIR-18-0637 PMC6445751

[153] F. Liu , J. Huang , F. He , X. Ma , F. Fan , M. Meng , Y. Zhuo , L. Zhang , Sci. Rep. 2020, 10, 10768. 32612110 10.1038/s41598-020-66806-z PMC7330044

[154] K. Wu , M. Yi , S. Qin , Q. Chu , X. Zheng , K. Wu , Exp. Hematol. Oncol. 2019, 8, 26. 31673481 10.1186/s40164-019-0150-0 PMC6815037

[155] Z. N. Willsmore , R. J. Harris , S. Crescioli , K. Hussein , H. Kakkassery , D. Thapa , A. Cheung , J. Chauhan , H. J. Bax , A. Chenoweth , R. Laddach , G. Osborn , A. McCraw , R. M. Hoffmann , M. Nakamura , J. L. Geh , A. MacKenzie‐Ross , C. Healy , S. Tsoka , J. F. Spicer , S. Papa , L. Barber , K. E. Lacy , S. N. Karagiannis , Front. Immunol. 2020, 11, 622442. 33569063 10.3389/fimmu.2020.622442 PMC7868381

[156] M.‐C. Veri , S. Burke , L. Huang , H. Li , S. Gorlatov , N. Tuaillon , G. J. Rainey , V. Ciccarone , T. Zhang , K. Shah , L. Jin , L. Ning , T. Minor , P. A. Moore , S. Koenig , S. Johnson , E. Bonvini , Arthritis Rheum. 2010, 62, 1933. 20506263 10.1002/art.27477

